# A Role for MeCP2 in Switching Gene Activity via Chromatin Unfolding and HP1γ Displacement

**DOI:** 10.1371/journal.pone.0069347

**Published:** 2013-07-23

**Authors:** Maartje C. Brink, Diewertje G. E. Piebes, Marloes L. de Groote, Martijn S. Luijsterburg, Corella S. Casas-Delucchi, Roel van Driel, Marianne G. Rots, M. Cristina Cardoso, Pernette J. Verschure

**Affiliations:** 1 Swammerdam Institute for Life Sciences, Netherlands Institute for Systems Biology, University of Amsterdam, Amsterdam, the Netherlands; 2 Department of Pathology and Medical Biology, University Medical Center Groningen, University of Groningen, Groningen The Netherlands; 3 Department of Biology, Technische Universitaet Darmstadt, Darmstadt, Germany; University of Insubria, Italy

## Abstract

Methyl-CpG-binding protein 2 (MeCP2) is generally considered to act as a transcriptional repressor, whereas recent studies suggest that MeCP2 is also involved in transcription activation. To gain insight into this dual function of MeCP2, we assessed the impact of MeCP2 on higher-order chromatin structure in living cells using mammalian cell systems harbouring a lactose operator and reporter gene-containing chromosomal domain to assess the effect of lactose repressor-tagged MeCP2 (and separate MeCP2 domains) binding in living cells. Our data reveal that targeted binding of MeCP2 elicits extensive chromatin unfolding. MeCP2-induced chromatin unfolding is triggered independently of the methyl-cytosine-binding domain. Interestingly, MeCP2 binding triggers the loss of HP1γ at the chromosomal domain and an increased HP1γ mobility, which is not observed for HP1α and HP1β. Surprisingly, MeCP2-induced chromatin unfolding is not associated with transcriptional activation. Our study suggests a novel role for MeCP2 in reorganizing chromatin to facilitate a switch in gene activity.

## Introduction

Gene activity is governed by the interplay between various proteins that modulate the epigenetic composition of chromatin (e.g. DNA methylation, histone modifications) [1]. Histone modifications and DNA methylation are linked by CpG-binding proteins such as methyl-CpG-binding protein 2 (MeCP2) [2] through, for instance, cross-talk between MeCP2 and heterochromatin protein 1 (HP1) isoforms [3]. MeCP2 is ubiquitously expressed in human tissues and particularly enriched at pericentromeric heterochromatin domains in brain cells [4], [5]. MeCP2 plays a role in neuronal maturation and impaired MeCP2 function results in neurodevelopmental disorders such as Rett syndrome [6], [7]. HP1 is a chromatin-binding protein that bridges H3K9-methylated histones with other chromatin-associated proteins thereby advancing the ‘spreading’ of heterochromatin [8], [9]. Both the clustering of pericentromeric heterochromatin domains and the relocalization of HP1 (in particular HP1γ) occur during myogenic differentiation when the level of methyl-CpG-binding proteins is up-regulated [3], [10].

MeCP2 was originally found to bind methylated DNA and to act as a transcriptional repressor [11]–[13]. More recent work demonstrated that MeCP2 also binds at actively transcribed genes and promotes activation of DNA-methylated genes, suggesting a role as a transcriptional activator [14]–[18]. Currently, MeCP2 is considered a multifunctional protein [19], i.e. MeCP2 is known (i) to bind methylated DNA [11], [12], [13], (ii) to recruit a wide range of proteins (e.g. chromatin-remodeling proteins Brahma, ATRX) [20]–[26], (iii) to induce the formation of repressive chromatin [5], [27], [28] and change the number and size of pericentromeric heterochromatin domains [29], (iv) to be involved in histone H1 displacement [21], [30]–[32], (v) to play a key role in neurological disease (e.g. Rett syndrome) involving both gene activation and repression [30], (vi) to be implicated in the regulation of imprinted genes [33]. To unambiguously assess how MeCP2 contributes to epigenetic gene regulation within the context of the mammalian genome, we targeted MeCP2, an MeCP2 Rett mutant (R133C) or separate MeCP2 domains as EGFP-lac repressor (lacR)-tagged fusions in cells harbouring a lac operator (lacO) and reporter gene-containing genomic domain [34]. Using this methodology, we previously showed that HP1 targeting is sufficient to induce local chromatin condensation and recruitment of histone methyltransferase SETDB1, concomitant with increased tri-methylation of H3K9 [35]. Here we show that MeCP2 targeting causes extensive chromatin decondensation of the targeted genomic domain, which occurs independently of the MeCP2 methyl-cytosine-binding domain (MBD) and results in eviction of the HP1γ isoform without an alteration in the transcriptional activity of the targeted chromatin.

## Materials and Methods

### Construction of plasmids

The full-length rat MeCP2e2 isoform and MeCP2 containing point mutation R133C [7] were PCR-amplified and cloned into the AscI site of p3'SS-EGFP-dimer *lac* repressor [36] resulting in C-terminally-tagged EGFP-lacR. Full-length MeCP2 or MBD, TRD or MBD-TRD domains were PCR-amplified and cloned into the XbaI and XhoI site of p3'SS-EGFP-dimer *lac* repressor, resulting in N-terminally-tagged EGFP-lacR. mCherry-lacR and mCherry-lacR-MeCP2 were created by excising EGFP from EGFP-lacR or EGFP-lacR-MeCP2 with XbaI and BsrGI followed by insertion of mCherry.

### Cell culture, transfection and luciferase reporter assay

Human osteosarcoma cells (U2OS) (ATCC 40342), NIH/3T3 mouse fibroblasts (ATCC, CRL-1658), AO3_1 and RRE_B1 clones (Andrew Belmont, University of Illinois, Urbana-Champaign (USA) [34]) and the U2OS 2-6-3 clone (David Spector, Cold Spring Harbor Laboratory, New York (USA) [35], [37]) were used. The AO3_1 and RRE_B1 clone are derivatives of CHO DG44 cells and contain an integrated amplified chromosomal region consisting of the dihydrofolate reductase (DHFR) cDNA transgene, 256 lac operator repeats and flanking DNA, representing a compact chromatin structure and an unusually extended fibrillar chromatin conformation, respectively. The 2-6-3 clone is a U2OS-derived clone containing a multicopy inducible transgene consisting of 256 lac operator repeats, a tetracycline-inducible reporter gene encoding cyano fluorescent protein with a peroxisomal targeting signal, 24 repeats of the MS2 bacteriophage translational operator, a splicing cassette and the 3’ UTR from the rabbit β globin gene [35], [37]. U2OS and NIH/3T3 cells were cultured in Dulbecco minimal essential medium containing 10% fetal bovine serum, 1% pen/strep. (Gibco) The AO3_1 and RRE_B1 clone were cultured in Ham’s F-12 medium without hypoxanthine or thymidine supplemented with 10% dialyzed fetal bovine serum (HyClone Labs, Logan, Utah), 1% pen/strep and methotrexate up to a final concentration of 0.03 μM or 10 μM, respectively. The 2-6-3 clone was cultured in high glucose Glutamax (Gibco) with 10% tetracycline free FBS (Clontech), 1% pen/strep and 100 μg/ml hygromycinB (Gibco). All cells were cultured at 37°C in a 5% CO_2_ atmosphere.

Transfection was performed with Lipofectamine 2000 (Invitrogen) or SAINT mix (Synvolux Therapeutics, Groningen, The Netherlands) in their respective media without Pen/Strep. For microscopy experiments cells were seeded on cover slips coated with Alcian Blue or 35 mm glass bottom dishes (MaTek). After 24–48 hours, cells were imaged directly or fixed in 4% paraformaldehyde for 15 minutes at 4°C and embedded in Vectashield (Brunschwig, Burlingame, CA) with DAPI (Vector Laboratories, Burlingame, CA).

Transfections for luciferase reporter assays were done with 8x lacO pGL3 luciferase vector, lacZ construct as an internal reference reporter and EGFP-lacR-tagged effector plasmids. Cells were harvested and lysed at 48 hours post-transfection. Luciferase reporter gene-targeted repression assay was performed as described previously [35]. Briefly, luciferase reporter assay transfections were done with 500 ng of the 8x lac operator containing pGL3 luciferase vector, 500 ng of pSV/β-Gal construct (Promega) as an internal reference reporter, and 500 ng of effector plasmid (EGFP-lacR, EGFP-lacR tagged full length MeCP2, C-terminus, ΔC-terminus and R133C) combined with 48 μl of FuGENE6 reagent per 25-cm^2^ culture flask. Cells were harvested and lysed at 48 h post-transfection and luciferase and β-Gal were detected.

### FACS sorting and quantitative PCR

Expressing cells were sorted by flow cytometry (Mo Flo XPD Cell sorter Beckman Coulter, Woerden, The Netherlands). Extracted mRNA was converted to cDNA using the Fermentas RevertAid^TM^ First strand cDNA synthesis kit. Quantitative PCR amplifications were performed on an ABI Prism 7900HT Sequence Detection System. All PCR reactions were carried out in triplicate using Taqman® gene expression assay Mm00515662_m1 for DHFR and Mm99999915_g1 for GapdH (Applied biosystems). Relative quantification of gene expression was calculated based on the comparative cycle threshold (Ct) method.

### BrUTP labeling, immunolabeling and fluorescent *in situ* hybridization

Nascent RNA run-on immunolabeling was performed as described previously [38], [39]. Briefly, cells were detergent permeabilized with 0.05% TritonX-100 (Sigma, Chemical Co.), and 10U/ml RNAsin in 20 mM Tris HCl, 0.5 mM MgCl2, 0.5 mM EGTA, 25% glycerin. For run-on transcription, the cells were incubated for 10 minutes in synthesis buffer, containing 0.5 mM BrUTP ATP, CTP and GTP. Subsequently, the cells were treated with 1mM PMSF and 5 U/ml RNAsin, fixed in 2% formaldehyde diluted in PBS and immunolabeled with rat anti BrdU (Seralab) diluted 1:500.

For immunolabeling 2% formaldehyde-fixed cells were treated with 0.5% TritonX-100 for 5 min, 100 mM Glycin for 10 minutes and blocked in 0.5% BSA. All treatments were buffered in 1xPBS. The primary antibodies were diluted in 1xPBS with 0.5% BSA and 0.05% Tween20. Primary antibodies include: rabbit anti-H3K9me2 (1:100) (Upstate, Milton Keynes, United Kingdom), rabbit anti-H3K9me3 (1:300) [40], rabbit anti-SETDB1 (1:200) [41], anti-EZH2 (1:1) and anti-EED(1:100) [42], rabbit anti-TFIIH p62 subunit (1:100) (SantaCruz Biotech), mouse anti-SC35 (1:1000) (Abcam), mouse anti-histone H1 (1:500) (Imgen), goat anti-hBrahma (N-19) (1:100) (Santa Cruz Biotechnology), rabbit anti-H3K4me2 (1:100) (Upstate), rabbit anti-H4K16ac (1:200), rabbit anti-H3K27me2 (Upstate), rat anti-PCNA (1:200) [43], [44], and rabbit anti-lacR (1:200) [36] and rabbit anti-MeCP2 (1: 500) [45]. For 5-methyl-cytosine labeling cells were fixed in 4% formaldehyde and treated as for regular immunolabeling in PBS, but also denatured in 2N HCl for 30 min at 37°C and blocked in 10% BSA prior to immunolabeling with mouse anti-5 mC (1:50) (Eurogentec) in 0.5% BSA and 0.05% Tween20, buffered in phosphate buffer pH7.4,

For fluorescence *in situ* hybridization, cells were fixed in 4% formaldehyde on ice, treated with 100 mM Glycin and 0.1N HCl, permeabilized with 0.5% Triton X-100 and 0.5% Saponin, buffered in 1xPBS [46]. Denaturation was buffered in SSC (0.15 M NaCl, 0.015 M sodium citrate) and carried out at 78°C in 2xSSC containing 50% formamide and 10% dextran sulfate. The probe was labeled following the instruction of the manufacture by nick translation with a DIG and biotine tag (Roche) Hybridization of the lac operator octamer probe occurred overnight at 37°C in deionized formamide with 0.1x COT DNA and 0.05x Sonicated Salmon sperm (Roche). The cells and probe (25 ng) were denatured and hybridized in 50% formamide and 10% dextran sulfate. Posthybridization washes were carried out with 2× SSC-50% formamide at 45°C. Probe detection proceeded at room temperature in 4× SSC containing 5% (w/v) nonfat dried milk. Antibodies used to detect DIG and biotine tagged probes are FITC-conjugated streptavidin, mouse anti-DIG. donkey anti-rabbit and donkey anti-mouse labeled with Cy3 or Cy5. All slides were stained with DAPI and embedded in vectashield.

### Image acquisition

Cells were imaged using a Zeiss LSM 510 (Zeiss, Jena, Germany) confocal laser scanning microscope equipped with a Zeiss Plan-Apochromat 63×/1.4 oil immersion objective or a Zeiss plan neofluar 63×/1.25 NA oil objective. We used multitrack scanning, employing a UV laser (364 nm), an argon laser (488 and 514 nm) and a helium-neon laser (543 nm) to excite DAPI staining and green/yellow and red fluorochromes. Emitted fluorescence was detected with BP 385-470, BP 505–550 and 560 LP filters. Three-dimensional (3D) images were scanned at 512 by 512 pixels averaging 4 times using a voxel size of 200 nm axial and 60 nm lateral.

To detect the fluorescence intensity levels of the endogenously immunolabeled MeCP2 and exogenous transfected fluorescently tagged MeCP2, a tile scan was made of 64 tiles of 512 by 512 pixels/tile.

### Image analysis

For quantitative analyses approximately 30 nuclei were imaged with comparable microscopical set-up. To quantitatively analyze changes in large-scale chromatin structure, we applied 3D image analysis tools (the Huygens system 2 software package; Scientific Volume Imaging, Hilversum, The Netherlands) as described previously [35], [47]. Briefly, 3D images of the amplified chromosome region are acquired after which the EGFP-lacR- labeled chromosome region is automatically identified. Specific features of the LacO array (3D structure, volume and intensity) are subsequently calculated using Huygens software. To assess the 3D chromatin structure, we used a parameter termed the surface factor, which represents the surface of a given chromosomal domain/object normalized to the surface of a sphere with an equal volume [43]. A surface factor of 1 therefore represents a perfectly spherical structure, whereas a lower value represents a more furrowed structure, such as a decondensed chromatin domain. The distribution of the calculated surface factors (∼30 nuclei per variable) is plotted in a box-plot. The second and third quartiles of the surface factor values are within the box, the median value is shown by the thick horizontal line, the vertical small lines show the first and fourth quartiles of the observed values. Since our surface factor data is not normally distributed (as tested with Shapiro Wilktest rejecting the null hypothesis that the data are normally distributed with p<0.05, p values see S1) and exhibits an unequal variance (Variance Equivalence test), we used the Wilcoxon nonparametric test [48] corrected for multiple testing (Bonferroni, p = 0.07) to calculate the P-value giving the probability that the control population (cells expressing EGFP-lacR) and the test population (cells expressing EGFP-lacR tagged MeCP2, separate MeCP2 domains or VP16 protein) are significantly different from each other. For the expression level measurements, the intensity of the transfected constructs at the lacO array was detected and normalized to the gain and offset settings of the PMT using a standard curve for the used parameters. The Pearson correlation coefficient was calculated to detect the correlation between the surface factor and the relative normalized intensities.

To depict variable expression levels of fluorescently tagged MeCP2, expression levels were measured using imageJ on both single scanned cells as well as tile-scanned images. Cells were masked and the nuclear counterstain intensities normalized.

### Photobleaching experiments

For FLIP and FRAP experiments, microscopes were equipped with an objective heater and cells were examined in microscopy medium (137 mM NaCl, 5.4 mM KCl, 1,8 mM CaCl_2_, 0.8 mM MgSO_4_, 20 mM D-glucose and 20 mM HEPES) at 37°C. FRAP and FLIP analysis was performed as described previously [49]. FRAP was used to measure the mobility of GFP-HP1γin- and outside of the array visualized by mCherry-LacR. Briefly, images were taken at 512×512 pixels (0.04×0.04 µm), 1.60 μs per frame, zoom 7. After 10 images, a square of 56×56 pixels was bleached for 10 scans (total time  = 1.1 s) and recovery was measured for at least 60 images at a 2-second time interval. The data was normalized to the original intensity before the bleach pulse by using the equation: I_FRAP_  = (I_strip t = t_ – I_background__t = t_)/(I_strip t = 0_ – I_background__t = 0_), where I_strip t = t_ and I_strip t = 0_ represent the intensity within the strip at t = t and the intensity before the bleach pulse (t = 0), respectively. For graphical representation, recovery plots were normalized between 0 and 1. FLIP analysis was used to measure the residence times of GFP-HP1γ on chromatin. Briefly, images of 512×512 pixels were acquired with a scan time of 1.60 μs (1x average/frame) at zoom 7 (1 pixel is 0.04×0.04 µm). After 10 images, a region of 275×150 pixels, occupying an area of 1/3 of the nucleus (excluding the lac operator array), was continuously bleached with maximal 488 nm and 514 nm laser intensity (AOTF 100%). EGFP-HP1γ fluorescence was monitored with low laser intensity for at least 80 images with a 2-second time interval between images. The loss of fluorescence in the unbleached part of the nucleus was quantified. All values were background corrected and normalized to 1 by using the equation: I_FLIP_  = (I_spot t = t_ - I_background t = t_)/(I_spot t = 0_ - I_background t = 0_). Curve fitting was performed according to N1*e^(λ1*t)^+N2*e^(λ·2*t)^.

## Results

### MeCP2 targeting causes local chromatin unfolding

To investigate the effect of MeCP2 targeting on chromatin folding we used cell lines that enable targeting of (EGFP-lacR-tagged) MeCP2 and separate MeCP2 domains to an integrated lacO genomic region, that is present in a highly amplified chromosomal domain in hamster cells (the AO3_1 and RRE_B1 clones) or as a multicopy genomic integration in human cells (the 2-6-3 clone). While the AO3_1, RRE_B1 as well as the 2-6-3 cells allow measurements of visual changes in 3-D chromatin folding upon targeting to the lacO array, it should be noted that the AO3_1 and RRE_B1 cells harbor a much larger array (80 Mpb) [35], [36], [50], [51] compared to the 2-6-3 cells (4 Mbp). Therefore, the impact of induced chromatin changes in 3-D folding are more striking in the AO3_1 and RRE_B1 cells. Importantly, the 2-6-3 clone exhibits less genomic instability then the AO3_1 and RRE_B1 clones, which makes it more suitable to study cell-cycle-dependent chromatin folding. Moreover, the 2-6-3 clone harbors a reporter gene containing 24 tandem MS2 repeats allowing visualization and measurement of changes in the transcript levels using YFP-tagged MS2 coat protein.

**Figure 1:**
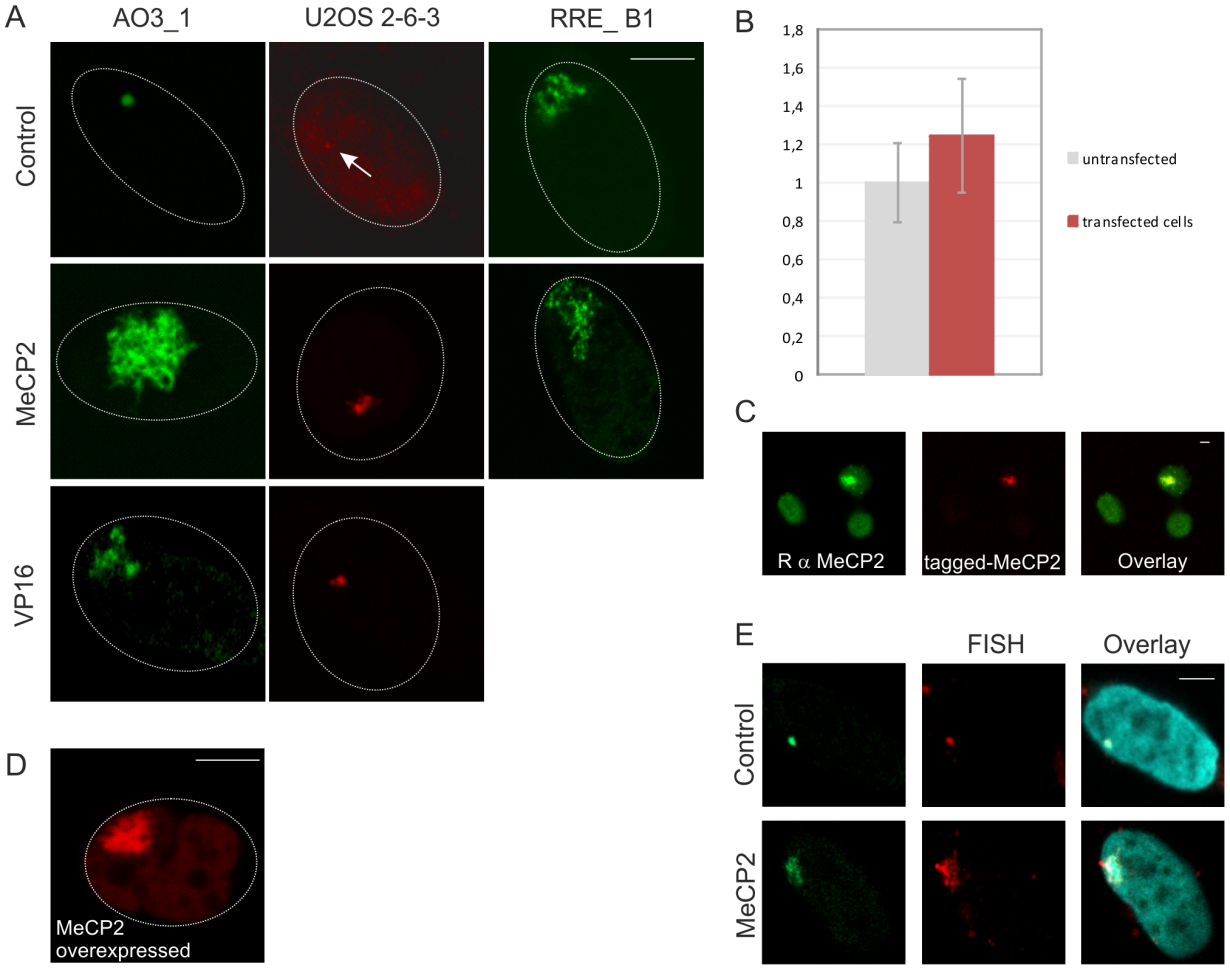
MeCP2 unfolds chromatin. The effect of MeCP2 lacR-lacO targeting on 3D chromatin folding was measured in AO3_1 and RRE_B1 clones (CHO derived, containing an amplified chromosomal region consisting of DHFR cDNA transgene, 256 lac operator repeats and flanking DNA) and the 2-6-3 clone (U2OS derived, containing lacO repeats and a tetracycline inducible reporter gene encoding cyano fluorescent protein and 24 repeats of the MS2 bacteriophage translational operator). The images show a typical representation of cells 48 hours after transfection. We imaged at least 100 cells per transfection and for quantitative measurents approximately 30 cells per transfection were imaged under comparable microscopical set-up (see Figure 4). (A) The images show individual optical sections of AO3_1 cells transfected with EGFP-lacR (control), EGFP-lacR-tagged MeCP2 or VP16, 2-6-3 cells transfected with EGFP-lacR (control), mCherry-lacR tagged MeCP2 or VP16 and RRE_B1 cells transfected with EGFP-lacR (control) or EGFP-lacR-tagged MeCP2. (B) AO3_1 cells expressing mCherry-lacR-tagged MeCP2 (n = 138) (shown in C) contain ∼25% more MeCP2 levels then endogenous MeCP2 (in non-transfected cells, n = 249). The error bars show the standard deviation of the analyzed cells (C) The images (a thick slice taken with open pinhole setting) to quantify endogenous/exogenous MeCP2 levels (shown in B), show AO3_1 cells transfected with mCherry-lacR tagged MeCP2 and immunolabeled with an antibody against MeCP2. (D) The images show an individual optical section of an AO3_1 cell highly over-expressing tagged MeCP2 (5,3 times higher than representative cells shown in (A). Representative cells are the cells that allow the Argos image analysis programme to select the lacO array from nuclear background, i.e. the cells on which we performed our quantitative measurements (see Figure 4 and S1). This analysis of MeCP2 over-expression illustrates that in cells exhibiting overexpressed MeCP2 levels a visually unfolded lacO chromatin array is observed similar as in the cells expressing representative MeCP2 levels. (E) The images show AO3_1 cells transfected with EGFP-lacR (control) and EGFP-lacR tagged MeCP2 (green signal), FISH-labeled with a fluorescent lacO probe (red signal) and DAPI-stained (blue signal). The images represent individual optical sections. Bars  = 5 μm.

We generated EGFP-lacR-tagged full-length MeCP2 or separate MeCP2 domain fusion proteins and expressed these fusions in the different cell lines harboring lacO arrays. Analysis of 3-D confocal images revealed that MeCP2 targeting induced extensive unfolding of the lacO array compared to targeting lacR in AO3_1, RRE_B1 and 2-6-3 cells (**Figure 1A** **)**. MeCP2 targeting induced unfolding of the lacO array in AO3_1 and 2-6-3 cells to the same extent as targeting of the viral activator VP16, which is known to cause extensive chromatin unfolding (**Figure 1A**) [35], [36], [50], [51]. We measured the expression level of exogenously expressed MeCP2 compared to endogenous MeCP2 to verify the effect of that physiological levels of MeCP2 can induce chromatin unfolding. Fluorescent immunolabeling using MeCP2 specific antibodies that recognize both exogenous and endogenous MeCP2 showed that mCherry-lacR tagged MeCP2 contained a ∼25% higher MeCP2 level compared to the endogenous MeCP2 level in non-transfected cells (**Figure 1B, C**). Moreover, we determined the effect of over-expression of exogenous MeCP2 on chromatin folding. This analysis shows that cells with highly over-expressed MeCP2 levels visually exhibit a comparable level of chromatin unfolding (**Figure 1D**) compared with cells that express intermediate levels of lacR-MeCP2 ((**Figure 1B, C**).

FISH labeling of the lacO array in AO3_1 cells using lacO probes confirmed that the MeCP2-induced unfolded structure overlapped completely with the unfolded lacO array (**Figure 1E**), showing that the EGFP-lacR visualized unfolded structure resembles the decondensed lacO array. Since the lacO chromosomal domain exhibits an extended fibrillar chromatin conformation in RRE_B1 cells and a compact chromatin (heterochromatic) structure in the AO3_1 and 2-6-3 cells, we used the AO3_1 and 2-6-3 cells for investigation of the effect of MeCP2 on epigenetic regulation [35], [47].

**Figure 2:**
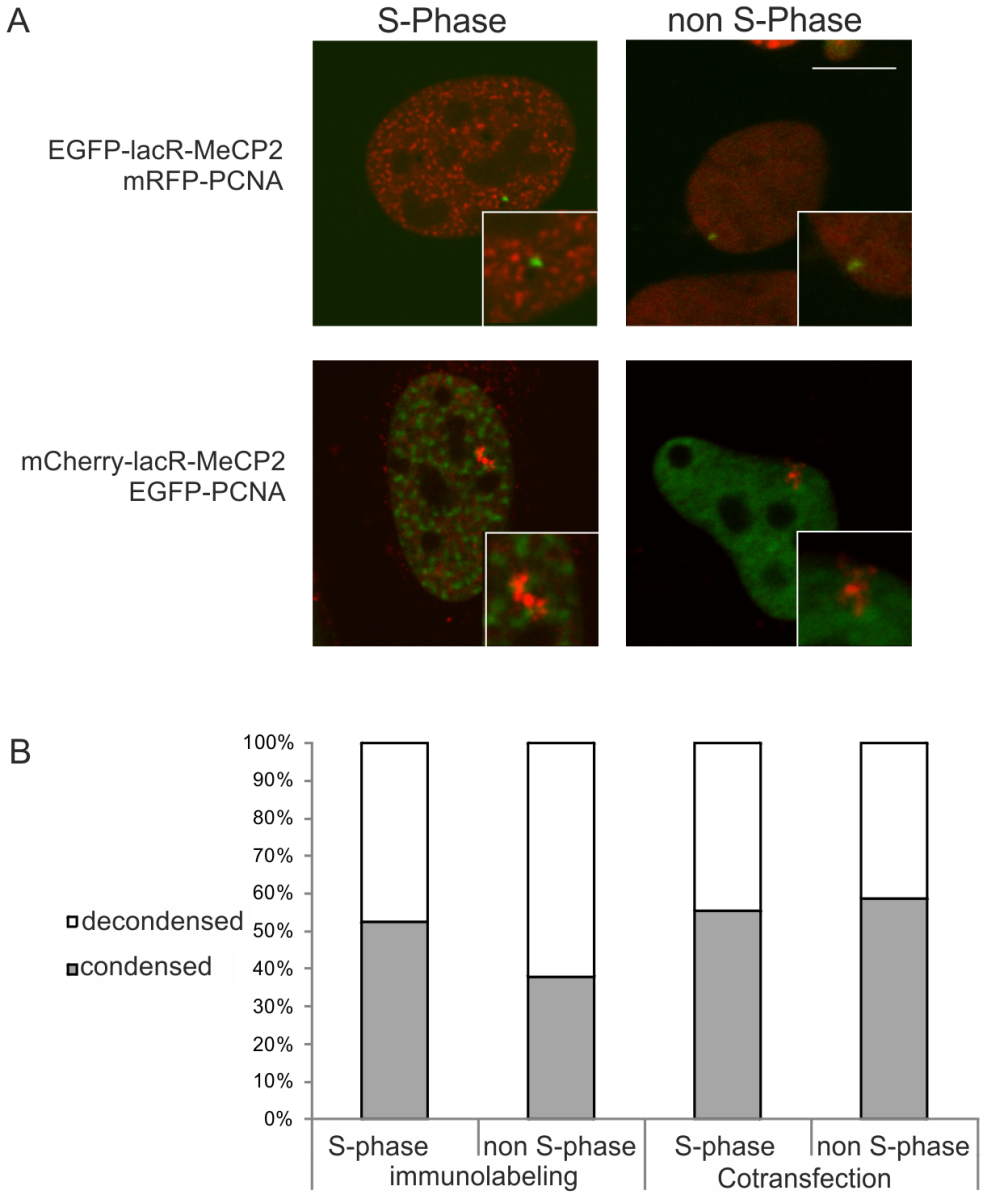
MeCP2-induced chromatin unfolding is independent of cell-cycle stage. (A) The images show 2-6-3 cells (U2OS derived clone containing a 200 copy chromosomal array of 256 lacO repeats and a reporter gene harboring 24 repeats of the MS2 bacteriophage) that were co-transfected with mCherry-tagged (red signal) or EGFP-tagged (green signal) lacR-MeCP2 and EGFP-tagged (green signal) or mRFP-tagged (red signal) PCNA. PCNA localizes at replication foci during S phase. The images represent individual optical sections of fixed cells. Bar  = 5 μm. (B) The histogram shows quantification of the number of lacR-MeCP2 transfected cells showing a condensed (grey bar) or decondensed (white bar) chromosomal array in either S or non-S phase based on PCNA expression or immunolabeling (on X-axis noted as immunolabeling, n = 74 and cotransfection, n = 68). A χ^2^ test was performed on PCNA immunolabeled (p = 0.25) and cotransfected cells (p = 0.81). Random variation probabilities show a high random variation between the decondensation and the cell cycle phase.

To study whether MeCP2-induced chromatin unfolding is cell-cycle dependent, we analyzed cells in S-phase based on the known accumulation of proliferating cell nuclear antigen (PCNA) into foci in replicating cells. These experiments were performed in human U2OS 2-6-3 cells containing a 200-copy lacO array [37]. Our data show that MeCP2-induced unfolding occurred as frequently in S-phase cells as it did in non S-phase cells based on transfections with mCherry-PCNA or staining for endogenous PCNA. These results illustrate that the MeCP2-induced chromatin structural changes are independent of the cell-cycle stage (**Figure 2A, B**).[44].

### MeCP2 chromatin unfolding acts independently of the MBD domain

**Figure 3:**
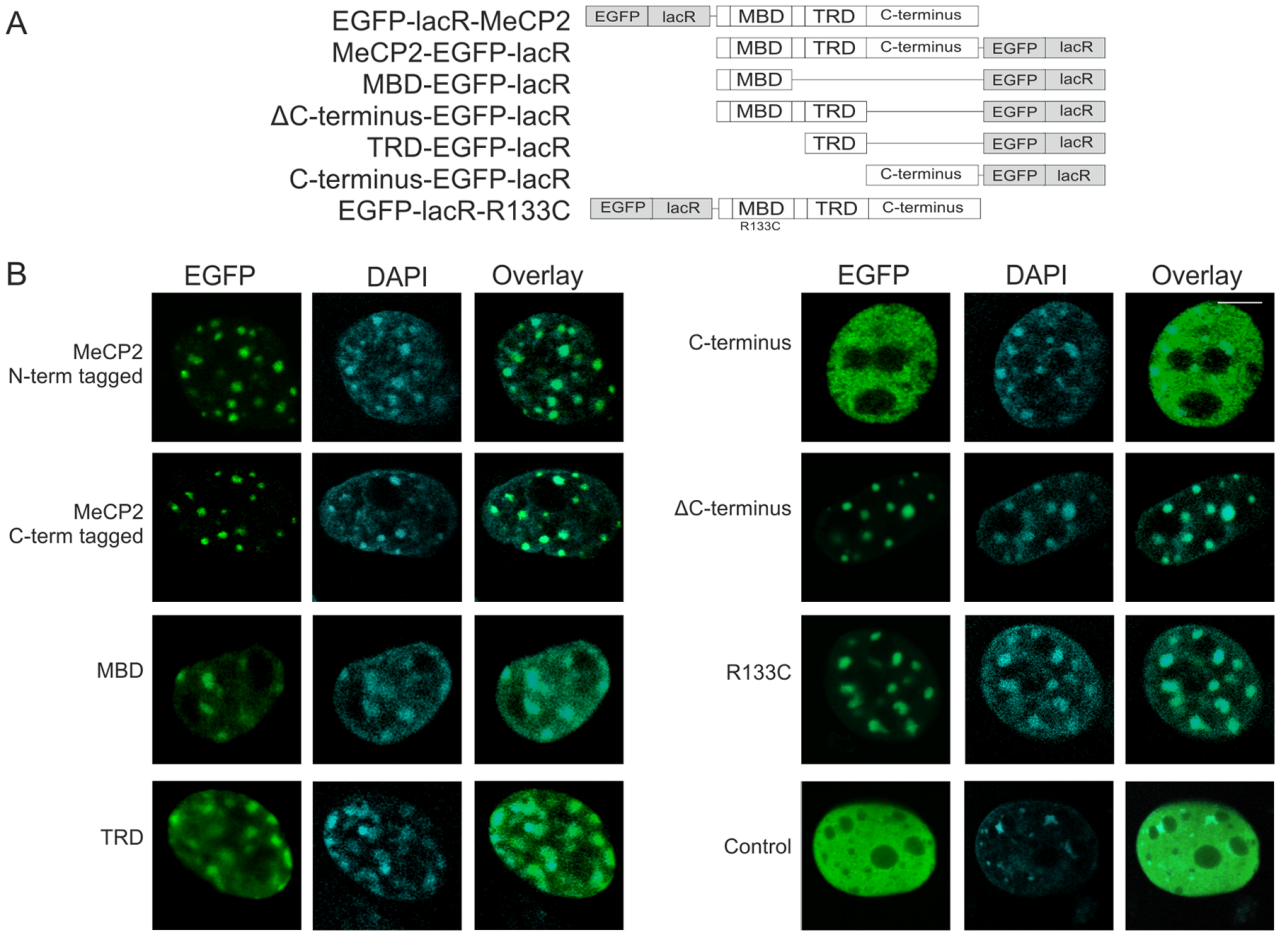
Nuclear distribution of EGFP-lacR-tagged MeCP2 domains in mouse fibroblasts. (A) The illustration shows a schematic representation of the tested constructs: EGFP-lacR tagged (grey boxes) full-length MeCP2, C- and N- terminally-tagged, separate MeCP2 domains (MBD, ΔC-terminus, TRD, C-terminus) and R133C Rett syndrome mutation (white boxes). (B) The images show EGFP-lacR-tagged MeCP2 and separate MeCP2 domains (green signal) that were expressed for 48 hours in NIH/3T3 cells and stained with DAPI (blue signal). MeCP2 and separate MeCP2 domains localize at DAPI dense chromocenters, except for the ΔC-terminus. We imaged approximately 100 cells per condition. The images represent individual optical sections of DAPI stained cells. Bar  = 5 μm.

**Figure 4:**
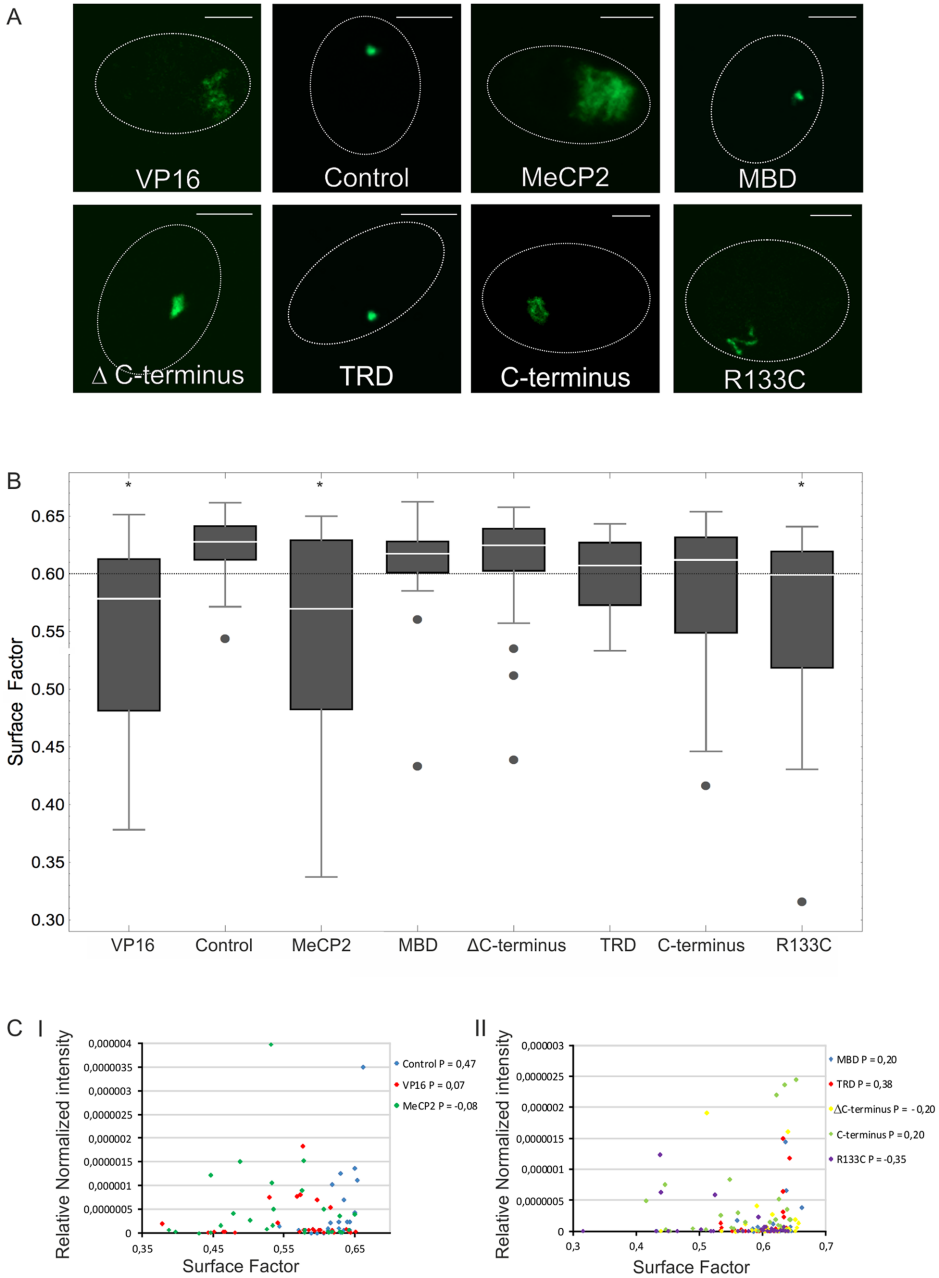
MeCP2-induced chromatin unfolding acts independently of the methyl-cytosine-binding domain. (A) The images show AO3_1 cells (CHO-derived clone containing an amplified chromosomal region consisting of the DHFR cDNA transgene and 256 lac operator repeats) that were transfected (green signal) with EGFP-lacR (control) or EGFP-lacR-tagged full-length MeCP2, -VP16 and -MeCP2 separate domains (i.e. MBD, ΔC-terminus, TRD, C-terminus and R133C Rett syndrome mutation). The images show a typical representation of cells transfected for 48 hours. For quantitative analysis 30 nuclei per condition were measured with comparable microscopical set-up. The images represent examples of individual optical sections. Bar  = 5 μm. (B) The figure shows the changes in lacO array large-scale chromatin structure measured with a 3D image analysis parameter, i.e. the surface factor. The surface factor determines the surface of a given chromosomal domain/object normalized to the surface of a sphere with an equal volume [43]. The distribution of the surface factor measurements is plotted as a box-plot. The second and third quartiles of the observed values are within the box, the median value is shown by the white horizontal line, the whiskers show the first and fourth quartiles of the observed values, dots are the outliers. The dotted line represents the ∼20% of cells in the EGFP-lacR control population that exhibit a mildly decondensed array (see also [69]). Considering a population (20%) of control cells exhibiting a decondensed configuration as the threshold of MeCP2-induced decondensation, the following percentages are found for lacR control 18%, VP16 68%, MeCP2 full length 47%, MBD 23%, ΔC-terminus 23%, C-terminus 47% and R133C 50%). We scored the chromatin structure based on the surface factor in the full cell population. Based on our statistical analysis the EGFP-lacR tagged VP16, MeCP2 (full length) and the R133C population are significantly different from EGFP-lacR control, whereas EGFP-lacR tagged MBD, TRD, C-terminus and ΔC-terminus, are not significantly different from EGFP-lacR (for p values see Table 1). (C) The figure shows the intensity of the transfected constructs measured within the lacO chromsomal array and plotted versus the chromatin surface factor measurements of the respective cells (I: Control, VP16 and MeCP2, II: MBD, TRD, C-terminus, ΔC-terminus and R133C). The Pearson correlation coefficient (Ρ) of the intensity of the transfected constructs and the determined surface factors are given.

MeCP2 harbors a methyl-binding domain (MBD), a transcription-repression domain (TRD) and a poorly characterized C-terminal domain [31], [52], [53]. We tagged various MeCP2 subdomains and regions spanning the MBD, TRD, the C-terminus and MeCP2 lacking its C-terminus (ΔC-terminus) or mutant MeCP2^R133C^ (Rett Syndrome point mutation in the MBD domain) [54] to EGFP-lacR to identify which MeCP2 subdomain is responsible for unfolding of the lacO chromosomal domain upon targeting (**Figure 3A**). First we tested the nuclear localization of the EGFP-lacR-tagged MeCP2 full-length and MeCP2 separate domain constructs in mouse fibroblasts lacking a lacO array. Similar to native full-length MeCP2, all lacR-tagged MeCP2 fusion proteins localized to pericentromeric heterochromatin in the mouse fibroblasts, except for the lacR-tagged C-terminus, which was homogeneously distributed in the nucleus (**Figure 3B** **)**. Next, we analyzed the effect of targeting EGFP-lacR tagged MeCP2 full-length, separate MeCP2 domains or VP16 versus EGFP-lacR (control) in the lacO-containing AO3_1 cells (**Figure 4**). Targeting of lacR-tagged MBD, TRD or ΔC-terminus domains did not cause chromatin unfolding into an extended fibrillar stucture as observed with full-length MeCP2 targeting, while targeting of MeCP2^R133C^ did result into mild chromatin structural changes. Targeting of the lacR-tagged MeCP2 C-terminus caused chromatin fibrillar unfolding but also to a much lesser extent than targeting of full-length MeCP2. **Figure 4A** shows typical representations of cells transfected for 48 hours with the respective constructs. For quantitative analysis approximately 30 nuclei per condition were imaged with a comparable microscopical set-up (**Figure 4B, C** **, Table S1**). We quantitatively assessed the change in chromatin structure by measuring the surface of the lacO chromatin domain relative to surface of a sphere with equal volume. A perfectly spherical structure, has a designated surface factor of 1, whereas a fibrillar unfolded chromatin structure will have a lower surface factor, due to its furrowed surface with equal volume. The quantitative measurements mirrored our visual observations: the degree of chromatin unfolding as observed in full-length MeCP2 (and VP16) was most pronounced in the cells targeted by separate MeCP2 domains containing the MeCP2 domains downstream of the MBD (i.e. MeCP2^R133C^ , C-terminus and TRD), while the MBD and ΔC-terminus show almost a similar chromatin structure as observed upon EGFP-lacR control targeting **(** **Figure** **4B** **,** **Table 1** **, Table S1)**. To verify whether the amount of expressed MeCP2 has an impact on chromatin unfolding we measured the fluorescence intensity of the transfected constructs in the lacO chromosomal array and we compared them with the surface factor measurements. Our intensity measurements show that there is no significant correlation between the amount of transfected construct and the extent of chromatin unfolding as measured by the surface factor (**Figure 4C** **, Table S1**). Our findings reveal that the MeCP2 regions downstream of the MBD are involved in MeCP2-induced chromatin unfolding whereas the MBD itself is dispensable for this phenomenon.

**Table 1:** Statistical evaluation of the chromatin structural analysis.

Populations analyzed	p-value
Control – VP16	p = 0.0000304522
Control – Full length MeCP2	p = 0.0000954561
Control – MeCP2^R133C^	p = 0.000273529
Control – MeCP2 C-terminus	p = 0.0125739
Control – MeCP2 TRD	p = 0.0135758
Control – MeCP2 MBD	p = 0.148108
Control – MeCP2ΔC-terminus	p = 0.346096

Statistical evaluation of the differences in chromatin structure after targeting EGFP-lacR tagged constructs. Rows 1 through 7 show the comparison between control cells transfected with EGFP-lacR and cells transfected with EGFP-lacR-tagged full-length MeCP2, EGFP-lacR-tagged VP16 and EGFP-lacR-tagged separate MeCP2 domains (i.e. C-terminus, MBD, TRD, ΔC-terminus, R133C Rett syndrome mutation). Since the data are not normally distributed and do not have a shared variance, we used Wilcoxon nonparametric statistical testing corrected for multiple testing (Bonferoni) [48]. The p-values are shown, indicating the probabilities that two populations are different choosing a cut-off value of p = 0.007. Based on this analysis the EGFP-lacR tagged VP16, MeCP2 (full length) and the R133C population are significantly different from EGFP-lacR control, whereas EGFP-lacR tagged MBD, TRD, C-terminus and ΔC-terminus, are not significantly different from EGFP-lacR.

### MeCP2-associated factors: MeCP2 targeting interferes with HP1γ binding

Through targeting to the lacO array in AO3_1 cells, we subsequently analyzed the accumulation or displacement of a variety of previously reported MeCP2-associated factors including proteins and epigenetic marks related to a transcriptionally active chromatin state (i.e. the Brahma subunit of the SWI/SNF complex [22], [24], [55], TFIIB, CREB1 [15], [56], RNA polymerase II, RNA splicing factor SC35 [57], H3K4 di-methylation and H4K16 acetylation and CpG methylation) (**Table 2**). While the distribution of most of these factors or epigenetic marks was not altered by targeting MeCP2, we confirmed that MeCP2 targeting interferes with chromatin binding of linker histone H1 (FRAP data not shown), which is compatible with the observed chromatin unfolding [19], [30]–[32]. Strikingly, we detected MeCP2-induced changes in the distribution of HP1γ and decided to study this in more detail.

**Table 2:** The presence of MeCP2 associated factors at the amplified chromosomal array upon MeCP2 targeting.

Labeling	control	MeCP2	Transfection	control	MeCP2
H3K9me2	+/−	+/−	H1	+/−	+/−
H3K9me3	+/−	+/−	RNAPII	−	−
H1	+/−	+/−	TFIIB	−	−
EZH2	+/−	+/−	CREB	−	−
EED	−	−	Dnmt1	+	+
TFIIHp62	−	−	Dnmt3b	−	−
hBrahma	−	−	HP1α	+	+
H3K4me2	−	−	HP1β	+	+
H4K16ac	−	−	HP1γ	+	−
H3K27me2	−	−			
SC35	−	−			
SETDB1	−	−			
mCpG	+	+			

Various factors were assayed at light microscopical level for their presence at the amplified chromosomal array in the AO3_1 clone (CHO derived clone containing an amplified chromosomal region consisting of the DHFR cDNA transgene and 256 lac operator repeats). Immunolabeling or co-transfection were performed upon expressing EGFP-lacR (control) or EGFP-lacR-MeCP2. Localization at the array is scored as (+) present, (+/−) infrequently present or (−) absent.

**Figure 5:**
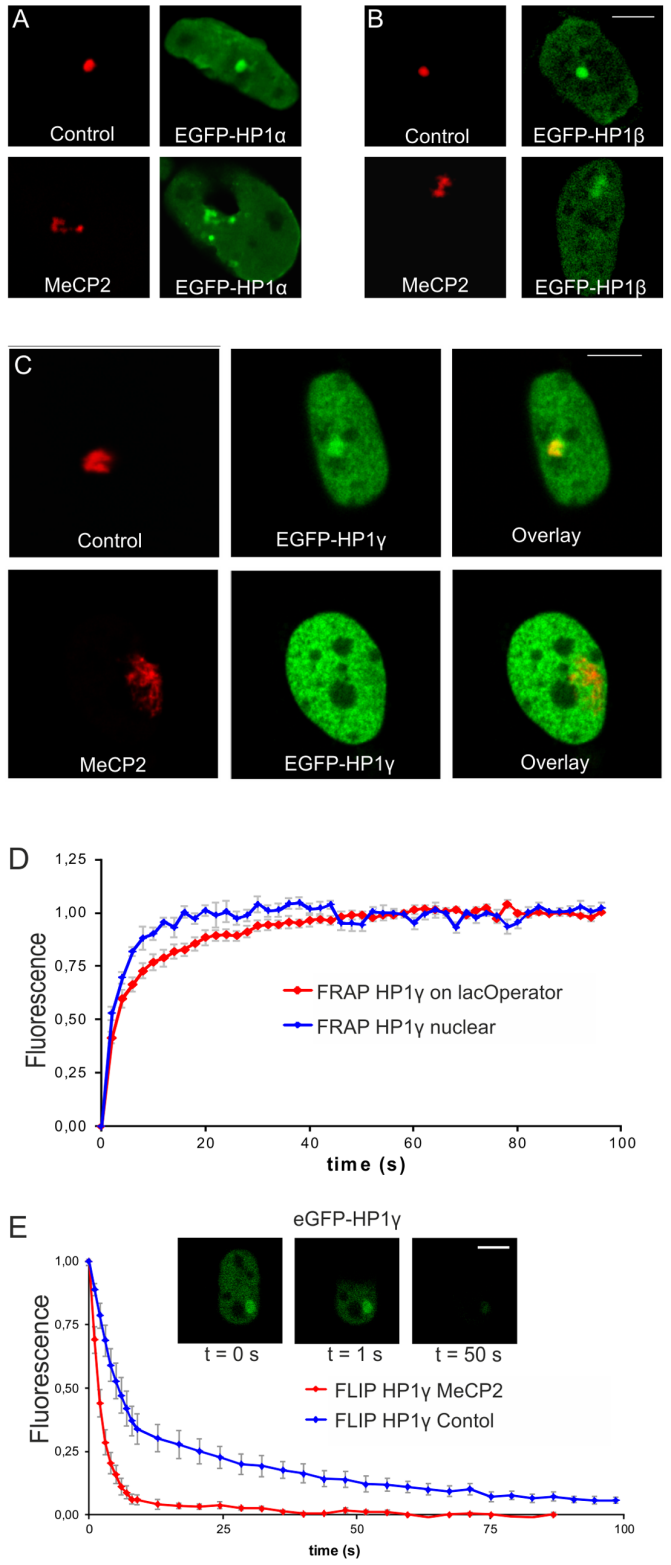
MeCP2 interferes with HP1γ binding. AO3_1 cells (CHO-derived clone containing an integrated lacO array) were co-transfected with EGFP-tagged HP1α, β or γ (green signal) and mCherry-tagged lacR or lacR-MeCP2 (red signal). (A-C) Pictures show 3D images that were recorded of living cells. The images represent individual optical sections and nuclei have the same scale. Bar  = 5 μm. (D) The curves show Fluorescent Recovery After Photobleaching (FRAP) data of HP1γ at the lac operator (red curve) as well as at the overall nuclear localization (blue curve) (E) The graphs show FLIP curves of EGFP-HP1γ in the presence of mCherry-lacR-MeCP2 (red line) or mCherry-lacR (blue line). The insets show an EGFP-HP1γ and mCherry-lacR targeted cell of which half of the nucleus is continuously bleached (only green channel is shown). Bar  = 5 μm. FLIP was measured in the bottom half of the nucleus.

We recently identified an interaction between MeCP2 and the HP1 proteins in mouse myoblast cells [3]. Co-transfection of mCherry-lacR and EGFP-tagged HP1α, β or γ in A03_1 cells showed enrichment of all HP1 isoforms at the lacO array (**Figure 5A–C**). Indeed, FRAP measurements on the mobility of HP1γ at the lacO array (visualized by mCherry-LacR) or elsewhere in the nucleus showed that HP1γ has a slower exchange rate at the array (**Figure 5D**). The measured binding kinetics of HP1γ at the lacO array is in accordance with previous findings on the binding dynamics of HP1 at pericentromeric heterochromatin [58]. Therefore, our findings imply that HP1γ binding at the lacO array in the AO3_1 cells reflects binding at heterochromatin, which is in agreement with the heterochromatic nature of the array **(** **Figure 5A–D** **)**. Strikingly, while HP1α and β remained bound **(** **Figure 5A, B** **)**, HP1γ accumulation at the lacO array was lost upon MeCP2 targeting (**Figure 5C**). We determined the HP1γ exchange rates using fluorescence loss in photobleaching (FLIP) **(** **Figure 5E** **)**, which identified two dynamic HP1γ pools corresponding to a freely diffusing (*t*_1/2_∼3 s) or a transiently chromatin-bound population (*t*_1/2_∼50 s) (**Figure 5E**). LacR-transfected cells displayed reduced HP1γ mobility at the array (37%, *t*_1/2_ = 52 s), indicating efficient retention of HP1γ at the array. Strikingly, the proportion of the freely diffusing HP1γ population went up to 97% (*t*_1/2 = _1.5 s) after targeting of lacR-tagged MeCP2. These results confirm that MeCP2 effectively antagonizes binding of HP1γ to chromatin.

**Figure 6:**
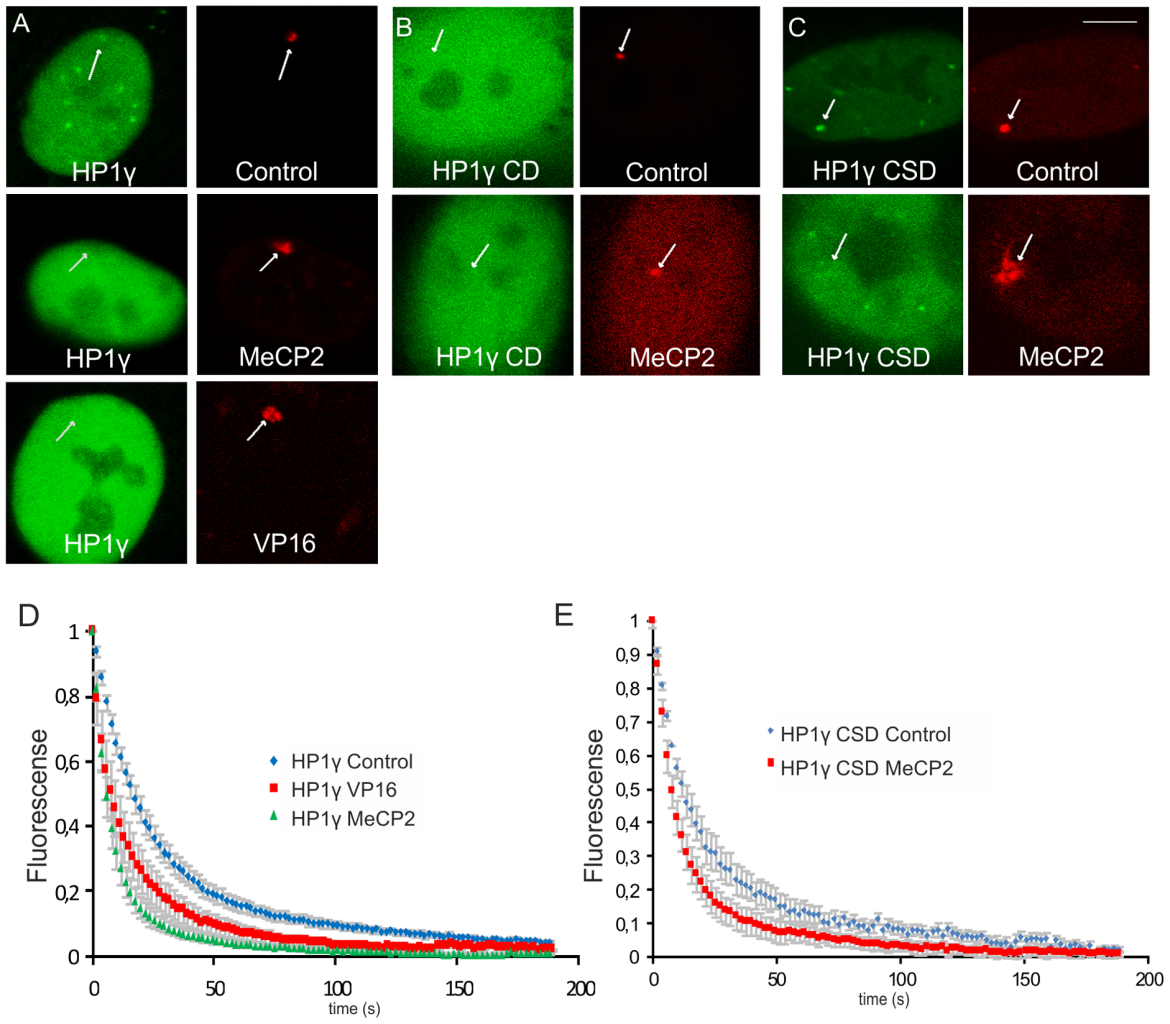
Interference with the binding of HP1γ and separate HP1γ domains. 2-6-3 clone (U2OS-derived clone containing a 200 copy chromosomal array of 256 lacO repeats and a reporter gene harboring 24 repeats of the MS2 bacteriophage) were co-transfected with YFP-tagged HP1γ, HP1γ CD (1–75) or HP1γ CSD (92–173) (green signal) and mCherry-tagged lacR, lacR-MeCP2 or lacR-VP16 (red signal). (A–C) Pictures show 3D images that were recorded of living cells (A–C). The images represent individual optical sections and nuclei have the same scale. Bar  = 5 μm. (D) The graphs show FLIP (Fluorescent loss after photobleaching) curves of YFP-HP1γ in the presence of mCherry-lacR (control, blue line), mCherry-lacR-MeCP2 (green line) or mCherry-lacR-VP16 (red line). (E) The graphs show FLIP curves of YFP-HP1γ CSD in the presence of mCherry-lacR (control, blue line) or mCherry-lacR-MeCP2 (red line).

To verify our observations that MeCP2 induces HP1γ dissociation from the lacO array in AO3_1 cells, we also analyzed this phenomenon in U2OS 2-6-3 cells (**Figure 6A–E**). Although the 200 copy lacO array in the 2-6-3 clone is smaller compared to the large amplified domain in the AO3_1 clone, we were able to confirm the MeCP2-induced HP1γ loss from the 200 copy lacO array in the 2-6-3 clone (**Figure 6A**). Similar to our analysis in AO3 cells, FLIP analysis in the 2-6-3 cells also revealed two dynamic HP1γ pools corresponding to freely diffusing (78% *t*_1/2_∼ 12.6 s) and chromatin-bound (22%, *t*_1/2_∼86.6 s) HP1γ pools. As in AO3 cells, MeCP2 targeting markedly shifted the proportion of freely diffusing HP1γ molecules towards 99.9 % in 2-6-3 cells, while 0.1% remained chromatin bound (**Figure 6D**). Similar to MeCP2 targeting, we found that the viral activator VP16 induced a comparable shift in HP1γ mobility in 2-6-3 cells (i.e. 98% freely diffusing and 2% chromatin-bound; **Figure 6A, D**).

To gain more insight into the MeCP2-induced HP1γdissociation from the lacO array upon chromatin unfolding, we analyzed the ability of MeCP2 to displace two separate HP1γ domains, the chromodomain, CD (1–75), which mediates HP1 binding to H3K9 tri-methylation, and the chromoshadow domain, CSD (92–173), which mediates HP1 dimerization and binding to a number of other proteins [59]. The HP1γ CSD domain localized at the lacO array upon targeting mCherry-tagged lacR whereas MeCP2 targeting triggered the displacement of the HP1γ CSD from the lacO array (**Figure 6B, C**). The distribution of the HP1γ CD was not affected upon MeCP2 targeting. FLIP analysis confirmed these findings and revealed that the mobility of the HP1γ CSD at the lacO array in control cells was similar to the full-length HP1γ as freely diffusing (73% *t*_1/2_∼ 8.77 s) and chromatin-bound (27%, *t*_1/2_∼57.8 s) pools could be detected. Upon targeting mCherry-lacR tagged MeCP2, a striking shift towards freely diffusing HP1γ CSD molecules (92%) could be measured, while only a small fraction remained chromatin bound (8%; **Figure 6E**). These experiments suggest that MeCP2-induced displacement of HP1γ is mediated through its CSD.

### MeCP2 targeting does not change the genomic transcriptional state

**Figure 7:**
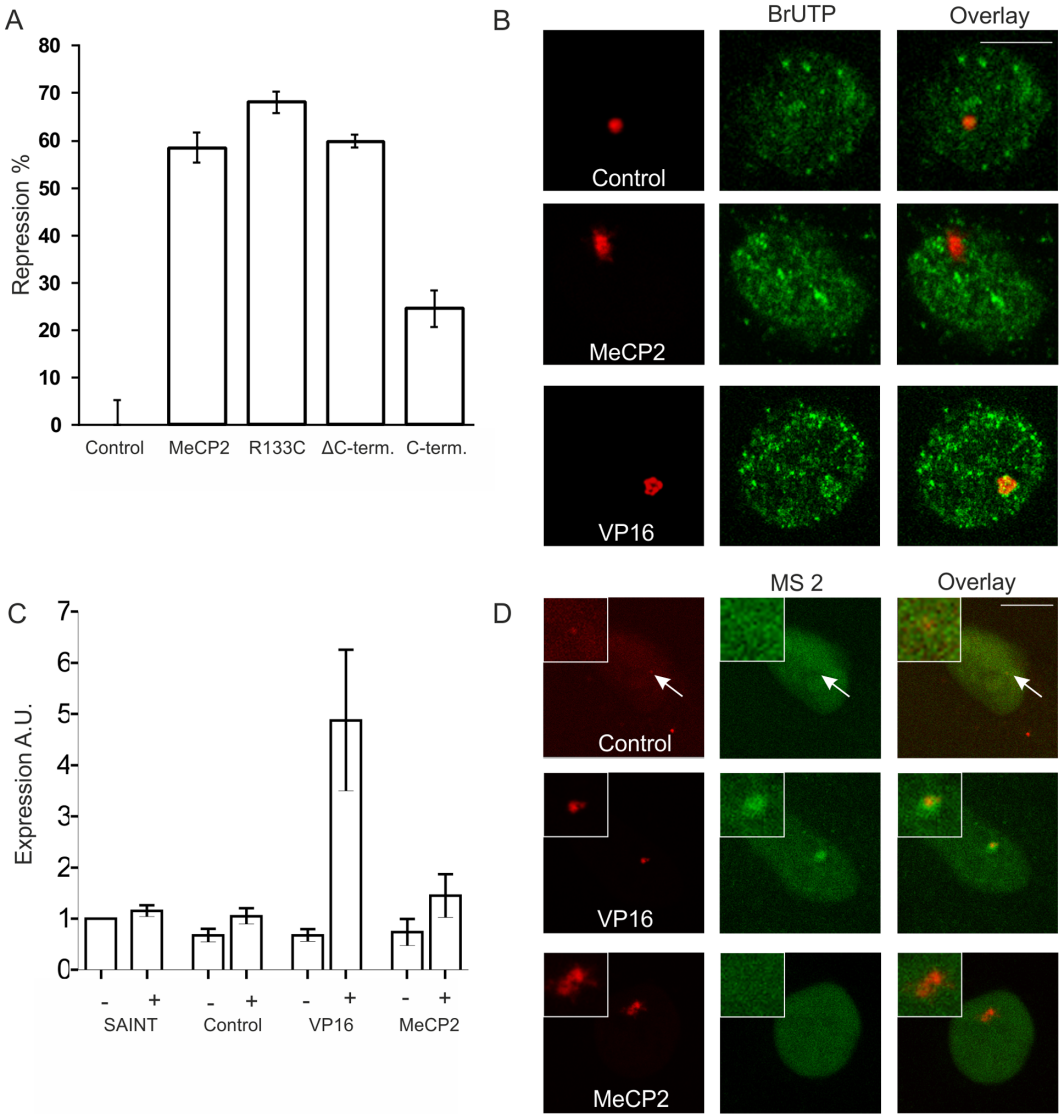
Reporter gene activity upon MeCP2-induced chromatin unfolding. (A) The histogram shows luciferase activity that was measured 48h after transfecting U2OS cells with β-Gal plasmid, an 8x lacO luciferase construct and the plasmids EGFP-lacR (control) and EGFP-lacR-tagged full length MeCP2 and separate MeCP2 domains (i.e. R133C Rett syndrome mutation, C-terminus and ΔC-terminus). Values are the mean ± standard error of 3 independent measurements. (B) AO3_1 cells (CHO-derived clone containing an amplified chromosomal region consisting of the DHFR cDNA transgene and 256 lac operator repeats) were transfected with mCherry-lacR, mCherry-lacR-MeCP2 or mCherry-lacR-VP16 (red signal) and nascent RNA was labeled by incorporation of BrUTP in permeabilized cells (green signal). Bar  = 5 μm. (C) The histogram shows DHFR transcriptional activity of AO3_1 cells that were transfected with EGFP-lacR (control), EGFP-lacR-MeCP2 or EGFP-lacR-VP16. Cells were sorted with the FACS and RT-qPCR was performed on both the EGFP-positive (+) as well as on the negative (−) cell population. Data was normalized to non-transfected samples and a transfection control was included (SAINT mix), which were both not FACS sorted. (D) The images show the 2-6-3 clone (U2OS-derived clone containing a 200 copy chromosomal array of 256 lacO repeats and a reporter gene harboring 24 repeats of the MS2 bacteriophage) that was transfected with MS2-YFP to visualize transcribed RNA (green signal) together with mCherry-LacR-tagged MeCP2 or VP16 (red signal detecting the lacO array). The images represent individual optical sections and nuclei are on the same scale. Bar  = 5 μm.

To test whether lacR-tagged MeCP2 and MeCP2 subdomains modulate gene activity at the promoter level, we measured gene expression levels of a transfected plasmid containing a luciferase reporter gene fused to 8 lacO copies [35]. Targeting LacR-tagged MeCP2, MeCP2^R133C^ and ΔC-terminus to a transiently expressed lacO-containing luciferase reporter gene in U2OS cells significantly repressed luciferase expression (60–70%) compared to targeting only lacR (**Figure 7A**). In contrast, targeting the C-terminal domain of MeCP2 caused repression to a moderate extent (∼25%) (**Figure 7A**). Taken together, these results demonstrate that the lacR-tagged MeCP2 protein is able to repress gene activity of a transiently expressed luciferase reporter gene plasmid. To address the effect of MeCP2 on gene activity at an integrated genomic locus, we assessed whether MeCP2 influences transcription of genes embedded in the lacO array integrated in the genome of AO3_1 cells. The MeCP2-induced unfolded chromatin structure in AO3_1 cells did not show enrichment of BrUTP-labeled transcripts from the DHFR selection gene compared to lacR-targeted control cells (**Figure 7B**). In contrast, a considerable increase in BrUTP-labeled transcripts was observed at the VP16-induced unfolded chromatin structure of the lacO chromosomal domain [50], [51] (**Figure 7B** **)**. Corroborating these findings, we performed RT-qPCR analysis of the DHFR reporter gene upon MeCP2 targeting and subsequent FACS sorting of the transfected AO3_1 cells. Our results show that the DHFR reporter gene expression was not significantly altered compared to lacR-targeted control cells, whereas VP16 targeting resulted in significantly enhanced expression levels of the reporter gene (**Figure 7C**). In addition, we employed the 2-6-3 clone to visualize MeCP2-induced changes in transcript levels [37]. Expression of MS2-YFP to visualize nascent transcripts showed that MeCP2 targeting did not activate transcription at the locus although significant unfolding of the chromosomal array was observed. Conversely, VP16 targeting induced significant accumulation of MS2-YFP-bound transcripts at the chromosomal array compared with MeCP2 targeting or lacR control targeting (**Figure 7D**). Taken together, we show that decondensation of the amplified chromosomal array upon MeCP2 targeting does not coincide with a change in gene activation.

## Discussion

Regulation of mammalian gene expression is a tightly controlled process. Mistakes in gene expression control can have far-reaching consequences, such as the manifestation of various developmental disorders or cancer. Mutations in the epigenetic regulatory protein MeCP2 underlies the developmental disorder known as Rett Syndrome [60]. Still, the molecular mechanisms underlying MeCP2-induced context-dependent functioning are largely unresolved. Post-translational MeCP2 modifications, changes in the MeCP2 genomic binding sites or regulation through MeCP2 co-factors likely influences whether MeCP2 acts as a transcriptional repressor or activator [60].

Here we provide evidence that direct targeting of MeCP2 as an EGFP-lacR tagged protein to a lacO-containing chromosomal domain induces extensive chromatin unfolding. Previous studies have shown that the lacR-lacO targeting system is a very powerful method to define the induced effect of (epigenetic) regulatory proteins on genomic behavior [35]–[38]. Engineered targeting systems allow to systematically unravel the cause-effect chain of the epigenetic state, chromatin folding, nuclear location and gene activity advancing our understanding of the principles of functional genome organization. We show that MeCP2 binding to native chromatin in intact cells triggers extensive chromatin unfolding and that the MBD is not required for this effect. In contrast to our findings, previous *in vitro* studies showed that binding of the C-terminal domain of MeCP2 to reconstituted nucleosomal arrays results in chromatin compaction [20], [28]. It should be noted that reconstituted nucleosomal arrays lack higher-order chromatin structure and could therefore respond differently to MeCP2 binding than chromatin embedded in the nucleus. In intact mouse myoblasts, overexpression of MeCP2 is shown to induce the MBD-dependent clustering of chromocenters during myogenic differentiation [10]. MeCP2 is a striking example of an intrinsically unstructured protein having a large number of regions predicted to acquire structure when complexed with binding partners [61]. The C-terminal portion of MeCP2 is known to be required for chromatin interactions, it harbors the Group II WW domain-binding motif required for splicing factor binding and the SPxK DNA-binding motif found in histone H1 [19]. Most likely, MeCP2 functioning depends on the type of chromatin and the MeCP2 domain involved. MeCP2 and histone H1 have been shown to compete for chromatin binding sites *in vitro* and *in vivo* [19], [32]. It has been suggested that a complex competitive equilibrium between MeCP2 and histone H1 for nucleosome and chromatin binding exists and that other competing chromatin binding proteins can affect this histone H1-MeCP2 binding equilibrium [21]. Such context-dependent *in vivo* functioning of MeCP2 is further underscored by a recent study demonstrating the unique physical properties and interaction domains of MeCP2 [21].

Several studies hint at a relationship between DNA methylation levels, the presence of methyl-CpG-binding proteins and changes in chromatin structure. For instance, a genome-wide loss of H3K9 di-methylation and a progressive increase in H3K9 acetylation, as well as increased chromocenter clustering was observed in mouse embryonic stem cells lacking DNA methyltransferases Dnmt3a and Dnmt3b. Moreover, during myogenic differentiation, overexpression of methyl-CpG-binding proteins induced increased DNA methylation levels and chromocenter clustering, independent of the H3K9 histone methylation pathway and requiring the MBD domain of MeCP2. We detected *in situ* CpG methylation both at the MeCP2-induced unfolded chromatin and the non-MeCP2 induced compact chromatin which is in line with our observations indicating that the chromatin unfolding is independent of the MeCP2 MBD domain.

Interestingly, we show that MeCP2 interferes with HP1γ chromatin binding. Our FRAP analysis shows similar kinetics of HP1γ at the lacO chromosomal domain as previously measured at mouse heterochromatic sites [58], [62]. The MeCP2-binding-induced release of HP1γ is reflected by the loss of local HP1γ accumulation at the lacO chromosomal domain. This MeCP2-induced interference with HP1γ is also observed with the HP1γ CSD but not with the HP1γ CD, indicating that the local (chromatin) protein composition influences the ability of MeCP2 to change the HP1γ binding kinetics. Moreover, we show that the transcriptional activator VP16 is also able to interfere with HP1γ binding upon chromatin unfolding and transcriptional activation, illustrating that the chromatin unfolding and HP1γ displacement is not restricted to the changes induced by MeCP2. However, while Janicki et al. showed that VP16-mediated unfolding triggered displacement of the HP1α isoform [37], we find MeCP2-induced chromatin unfolding to result in the selective removal of the HP1γ isoform. In breast cancer cells displacement of HP1γ is shown to precede transcriptional activation of an integrated luciferase reporter gene [63]. In this study, hormonal signaling triggered phosphorylation of H3S10, displacement of HP1γ and ATP-dependent chromatin remodeling resulting in an open, transcriptionally competent chromatin structure. It is tempting to speculate that the MeCP2-mediated chromatin unfolding and eviction of HP1γ are part of a similar mechanism to render chromatin amenable to subsequent transcriptional changes.

Our finding that MeCP2 mediates extensive chromatin unfolding, while maintaining transcriptional silencing of genes embedded within the unfolded chromatin structure, is quite surprising. Recent evidence suggests that proteins that typically accumulate at pericentromeric heterochromatin such as HP1 may function as transcriptional activators, in addition to their role as transcriptional silencer [18], [64], [65]. Such findings would argue that the canonical view in which open chromatin is transcriptionally active and closed chromatin is transcriptionally inert is too simplistic. Our data might indicate that MeCP2-induced chromatin unfolding prepares chromatin for subsequent transcriptional regulation. Examples of changes in chromatin structure preceding transcriptional activation have been reported for the *HoxB* and *MHC* locus [66], [67]. Moreover, biochemical analyses revealed that transcriptionally inactive sites can occur both in compact and less compact chromatin [68]. We propose that MeCP2-mediated chromatin unfolding reflects an indeterminate state that facilitates a switch in gene activity. In this scenario, the action of subsequent regulatory factors determines the transcriptional fate of genes embedded within chromatin that has been rendered permissive by MeCP2. Such a role of MeCP2 fits well with recent observations in neuronal cells, where MeCP2 is abundantly present and proposed to act as a versatile global transcriptional regulator in concert with other regulatory proteins [5]. Elucidating this global role of MeCP2 in restructuring chromatin *in vivo* is intriguing and may aid in understanding the pathophysiology of neurodevelopmental disorders, such as Rett syndrome.

## Supporting Information

Table S1 Quantitative data MeCP2-induced chromatin unfolding. Our measurements of the surface factor and the testing for a normal distribution of the surface factor data (Shapiro Wilktest), the microscopical gain and offset settings and the measurements of volume and intensity of the lacO chromosomal array are provided. AO3_1 cells (CHO-derived clone containing an amplified chromosomal region consisting of the DHFR cDNA transgene and 256 lac operator repeats) were transfected with EGFP-lacR (control) or EGFP-lacR-tagged full-length MeCP2, -VP16 and -MeCP2 domains (i.e. MBD, ΔC-terminus, TRD, C-terminus and R133C Rett syndrome mutation) and 30 nuclei per transfected construct were measured with comparable microscopical set-up. We applied a 3D image analysis parameter (the Huygens system 2 software package; Scientific Volume Imaging, Hilversum, The Netherlands) as described previously [35], [47]. Specific features of the LacO array (3D structure, volume and intensity) are calculated using Huygens software. The EGFP-lacR- labeled chromosome region is automatically identified in the acquired 3D images, given as the volume. Changes in lacO array large-scale chromatin structure are measured with a 3D image analysis parameter, i.e. the surface factor. The surface factor determines the surface of a given chromosomal domain/object normalized to the surface of a sphere with an equal volume [43]. We tested with Shapiro Wilktest whether the surface factor data is normally distributed. The intensity of the transfected constructs at the lacO array is detected, i.e. total array intensity and normalized to the gain and offset settings of the PMT using a standard curve for the used parameters thereby providing the relative normalized intensity of the transfected constructs.
(DOCX)
