# Supplementary material for: A Role for MeCP2 in Switching Gene Activity via Chromatin Unfolding and HP1γ Displacement
Source: PLoS One. 2013 Jul 23;8(7):e69347. doi: 10.1371/journal.pone.0069347 (PMC3720725; doi:10.1371/journal.pone.0069347)
Supplement: Table S1 — Quantitative data MeCP2-induced chromatin unfolding. Our measurements of the surface factor and the testing for a normal distribution of the surface factor data (Shapiro Wilktest), the microscopical gain and offset settings and the measurements of volume and intensity of the lacO chromosomal array are provided. AO3_1 cells (CHO-derived clone containing an amplified chromosomal region consisting of the DHFR cDNA transgene and 256 lac operator repeats) were transfected with EGFP-lacR (control) or EGFP-lacR-tagged full-length MeCP2, -VP16 and -MeCP2 domains (i.e. MBD, ΔC-terminus, TRD, C-terminus and R133C Rett syndrome mutation) and 30 nuclei per transfected construct were measured with comparable microscopical set-up. We applied a 3D image analysis parameter (the Huygens system 2 software package; Scientific Volume Imaging, Hilversum, The Netherlands) as described previously [35], [47]. Specific features of the LacO array (3D structure, volume and intensity) are calculated using Huygens software. The EGFP-lacR- labeled chromosome region is automatically identified in the acquired 3D images, given as the volume. Changes in lacO array large-scale chromatin structure are measured with a 3D image analysis parameter, i.e. the surface factor. The surface factor determines the surface of a given chromosomal domain/object normalized to the surface of a sphere with an equal volume [43]. We tested with Shapiro Wilktest whether the surface factor data is normally distributed. The intensity of the transfected constructs at the lacO array is detected, i.e. total array intensity and normalized to the gain and offset settings of the PMT using a standard curve for the used parameters thereby providing the relative normalized intensity of the transfected constructs. (DOCX) [file pone.0069347.s001.docx]

| **Control** | | | | | |
| --- | --- | --- | --- | --- | --- |
| **Shapiro Wilcox test of Surface factor measurements = 0,0434739** | | | | | |
| **Surface factor** | **Total array intensity** | **Volume** | **Gain** | **Offset** | **Relative normalised intensity** |
| 0,650467 | 8,04826E+12 | 8497180 | 514 | -0.060000 | 4,26466E-07 |
| 0,661559 | 1,53006E+13 | 8929937 | 464 | -0.060000 | 3,50924E-06 |
| 0,650289 | 1,89472E+13 | 7943669 | 497 | -0.060000 | 1,36626E-06 |
| 0,634596 | 7,04973E+12 | 3789071 | 517 | -0.060000 | 6,97712E-07 |
| 0,627942 | 6,91083E+12 | 5515530 | 572 | -0.060000 | 2,33621E-07 |
| 0,61632 | 1,64091E+12 | 2493411 | 547 | -0.060000 | 2,01186E-07 |
| 0,600762 | 1,41765E+12 | 2767556 | 637 | -0.060000 | 6,16517E-08 |
| 0,653096 | 1,10214E+13 | 6768763 | 484 | -0.060000 | 1,10229E-06 |
| 0,543377 | 1,83829E+13 | 17925799 | 682 | -0.060000 | 1,38058E-07 |
| 0,629544 | 1,08554E+13 | 5332767 | 485 | -0.060000 | 1,24473E-06 |
| 0,641315 | 7,70179E+12 | 10102884 | 605 | -0.060000 | 7,26326E-08 |
| 0,63474 | 2,20392E+12 | 2009742 | 585 | -0.060000 | 6,66604E-08 |
| 0,591621 | 8,17591E+12 | 11870465 | 670 | -0.060000 | 8,97718E-08 |
| 0,642855 | 3,42994E+12 | 6717850 | 690 | -0.060000 | 7,06838E-08 |
| 0,639964 | 3,00142E+12 | 3002537 | 543 | -0.050000 | 2,20514E-07 |
| 0,643123 | 6,07756E+12 | 3960085 | 549 | -0.060000 | 2,31194E-07 |
| 0,629743 | 1,45339E+12 | 1940553 | 605 | -0.060000 | 1,37064E-08 |
| 0,627603 | 1,27801E+13 | 10762790 | 583 | 0.000000 | 9,49638E-08 |
| 0,60674 | 2,30367E+12 | 7470443 | 817 | 0.000000 | 1,00712E-09 |
| 0,596647 | 2,36981E+12 | 6670854 | 817 | 0.000000 | 1,13522E-09 |
| 0,618516 | 1,71714E+13 | 8701483 | 536 | 0.000000 | 1,0267E-06 |
| 0,633644 | 8,58165E+12 | 8715843 | 586 | 0.000000 | 4,36866E-08 |
| 0,571435 | 7,96086E+12 | 9314392 | 656 | 0.000000 | 5,9074E-08 |
| 0,612126 | 5,96342E+12 | 8254366 | 666 | 0.000000 | 9,49125E-08 |
| 0,612557 | 1,50118E+13 | 10555876 | 568 | 0.000000 | 6,45869E-08 |
| 0,6153 | 4,98711E+12 | 4651321 | 573 | 0.000000 | 2,20064E-08 |
| 0,580172 | 6,78701E+12 | 9405773 | 648 | 0.000000 | 7,39899E-08 |
| 0,588705 | 2,69407E+12 | 4811239 | 1002 | 0.000000 | 1,70388E-09 |

| **VP16** | | | | | |
| --- | --- | --- | --- | --- | --- |
| **Shapiro Wilcox test of Surface factor measurements = 0,00526125** | | | | | |
| **Surface factor** | **Total array intensity** | **Volume** | **Gain** | **Offset** | **Relative normalised intensity** |
| 0,601949 | 9,07267E+12 | 13609978 | 719 | -0.045000 | 6,43221E-08 |
| 0,618604 | 8,35228E+12 | 9027193 | 664 | -0.045000 | 7,29604E-08 |
| 0,585675 | 7,39317E+12 | 9277839 | 734 | -0.045000 | 5,71351E-08 |
| 0,606812 | 6,56994E+12 | 9132282 | 659 | -0.045000 | 1,62974E-08 |
| 0,529452 | 4,72098E+13 | 43053770 | 615 | -0.035000 | 7,43412E-07 |
| 0,599711 | 3,05547E+13 | 20641789 | 771 | -0.045000 | 7,27693E-08 |
| 0,572987 | 5,05203E+13 | 30343900 | 581 | -0.045000 | 8,07967E-07 |
| 0,638454 | 9,4312E+12 | 13901746 | 636 | -0.045000 | 7,74314E-08 |
| 0,576877 | 3,32756E+13 | 26635767 | 530 | -0.045000 | 1,82842E-06 |
| 0,651225 | 3,78117E+12 | 4762937 | 600 | -0.045000 | 3,56555E-08 |
| 0,378191 | 2,51875E+13 | 65955294 | 650 | -0.045000 | 1,97078E-07 |
| 0,451477 | 1,00908E+13 | 15955220 | 720 | -0.045000 | 2,85448E-08 |
| 0,467441 | 8,1737E+12 | 25847274 | 788 | -0.045000 | 3,80878E-08 |
| 0,481229 | 2,82625E+12 | 11952708 | 838 | -0.045000 | 5,40931E-09 |
| 0,644142 | 1,25275E+12 | 2285844 | 675 | -0.045000 | 1,49004E-08 |
| 0,446855 | 3,87602E+12 | 11462511 | 790 | -0.045000 | 7,38259E-09 |
| 0,568126 | 3,27314E+13 | 26964740 | 543 | -0.045000 | 7,68328E-07 |
| 0,620049 | 1,62528E+12 | 4706150 | 796 | -0.045000 | 8,68969E-09 |
| 0,443408 | 3,06214E+12 | 7078807 | 722 | -0.045000 | 1,94636E-08 |
| 0,591027 | 5,18835E+12 | 9825476 | 677 | -0.045000 | 8,12077E-08 |
| 0,59752 | 3,19481E+13 | 31320377 | 582 | -0.045000 | 6,92818E-07 |
| 0,593633 | 4,81868E+12 | 9700153 | 707 | -0.045000 | 4,06453E-08 |
| 0,464881 | 1,2868E+13 | 17966920 | 844 | -0.045000 | 2,67565E-08 |
| 0,571665 | 5,67181E+12 | 11679869 | 695 | -0.045000 | 2,76353E-08 |
| 0,542003 | 3,61127E+13 | 30032550 | 590 | -0.045000 | 2,11536E-07 |

| **MeCP2** | | | | | |
| --- | --- | --- | --- | --- | --- |
| **Shapiro Wilcox test of Surface factor measurements = 0,0031525** | | | | | |
| **Surface factor** | **Total array intensity** | **Volume** | **Gain** | **Offset** | **Relative normalised intensity** |
| 0,578956 | 1,93063E+12 | 6495271 | 857 | -0.040000 | 6,1395E-09 |
| 0,612432 | 3,21228E+12 | 5441772 | 718 | -0.040000 | 2,38561E-08 |
| 0,578968 | 5,78629E+12 | 8409714 | 683 | -0.040000 | 6,80428E-08 |
| 0,502626 | 7,909E+12 | 13726816 | 588 | -0.047000 | 2,67206E-07 |
| 0,532572 | 1,12858E+14 | 75098019 | 556 | -0.047000 | 3,9634E-06 |
| 0,478765 | 6,7421E+12 | 6722419 | 556 | -0.047000 | 4,01567E-07 |
| 0,534254 | 1,65999E+13 | 14428496 | 691 | -0.047000 | 1,55186E-07 |
| 0,613157 | 4,3201E+12 | 5257051 | 777 | -0.047000 | 8,30854E-09 |
| 0,526184 | 8,39646E+12 | 12956600 | 692 | -0.047000 | 7,73566E-08 |
| 0,578614 | 2,43003E+13 | 18940787 | 531 | -0.040000 | 1,52586E-06 |
| 0,43035 | 1,66487E+12 | 10553265 | 918 | -0.040000 | 1,27859E-09 |
| 0,460854 | 3,27013E+13 | 25183453 | 568 | 0.000000 | 1,60544E-07 |
| 0,532744 | 2,67408E+13 | 21522316 | 563 | 0.000000 | 1,05096E-06 |
| 0,632565 | 2,81608E+12 | 3196396 | 890 | 0.000000 | 8,14082E-09 |
| 0,6283 | 1,27139E+13 | 8619892 | 605 | 0.000000 | 3,59876E-07 |
| 0,649869 | 2,48069E+13 | 11912892 | 560 | 0.000000 | 3,88448E-07 |
| 0,575816 | 1,55991E+13 | 12104793 | 525 | 0.000000 | 8,95493E-07 |
| 0,535294 | 2,8112E+13 | 34720424 | 665 | 0.000000 | 4,93996E-07 |
| 0,606278 | 1,25308E+13 | 9999754 | 570 | 0.000000 | 5,00541E-07 |
| 0,397216 | 7,31051E+12 | 11601542 | 749 | 0.000000 | 2,56189E-08 |
| 0,64209 | 2,85223E+12 | 4698970 | 654 | 0.000000 | 7,31357E-08 |
| 0,629167 | 8,90113E+12 | 6946957 | 779 | 0.000000 | 3,38623E-08 |
| 0,618142 | 3,59784E+12 | 4695054 | 664 | 0.000000 | 3,83295E-08 |
| 0,488627 | 5,07777E+13 | 43050506 | 579 | 0.000000 | 1,5023E-06 |
| 0,387532 | 4,85031E+12 | 8570285 | 692 | 0.000000 | 6,018E-08 |
| 0,446503 | 5,03416E+12 | 6339269 | 822 | 0.000000 | 1,81676E-08 |
| 0,629695 | 1,40282E+12 | 2153994 | 652 | 0.000000 | 3,78542E-08 |
| 0,447096 | 5,58983E+13 | 46719476 | 592 | 0.000000 | 1,22577E-06 |
| 0,638161 | 4,29339E+12 | 4502500 | 652 | 0.000000 | 7,9016E-08 |
| 0,642385 | 7,16678E+12 | 6783123 | 674 | 0.000000 | 8,0388E-08 |
| 0,5103 | 8,76029E+12 | 8817668 | 624 | 0.000000 | 1,6673E-08 |
| 0,645305 | 4,86703E+12 | 4484224 | 834 | 0.000000 | 4,09015E-09 |

| **MBD** | | | | | |
| --- | --- | --- | --- | --- | --- |
| **Shapiro Wilcox test of Surface factor measurements = 1,71773E-06** | | | | | |
| **Surface factor** | **Total array intensity** | **Volume** | **Gain** | **Offset** | **Relative normalised intensity** |
| 0,619164 | 6,76064E+12 | 14291423 | 772 | 0.000000 | 1,89128E-08 |
| 0,601635 | 5,66361E+12 | 10209931 | 697 | 0.000000 | 1,97246E-08 |
| 0,611791 | 4,50684E+12 | 6851006 | 702 | 0.000000 | 1,25769E-08 |
| 0,620429 | 2,44924E+12 | 4259033 | 687 | 0.000000 | 1,25564E-08 |
| 0,620041 | 2,07013E+12 | 4783172 | 752 | 0.000000 | 1,59428E-08 |
| 0,627839 | 2,29662E+12 | 4817766 | 752 | 0.000000 | 9,84974E-09 |
| 0,624398 | 2,151E+12 | 4826252 | 767 | 0.000000 | 8,70124E-09 |
| 0,432889 | 6,65439E+12 | 14579928 | 737 | 0.000000 | 1,34218E-08 |
| 0,637365 | 9,09277E+12 | 6096456 | 535 | 0.000000 | 6,53429E-07 |
| 0,596726 | 2,7192E+12 | 5153267 | 687 | 0.000000 | 3,03326E-08 |
| 0,585121 | 2,73086E+12 | 4363470 | 687 | 0.000000 | 5,53887E-08 |
| 0,617437 | 3,97933E+12 | 4734218 | 592 | 0.000000 | 1,21883E-07 |
| 0,64489 | 6,27164E+12 | 5154573 | 507 | 0.000000 | 7,72767E-08 |
| 0,611391 | 2,93658E+12 | 5274022 | 670 | 0.000000 | 1,07829E-08 |
| 0,606442 | 5,30355E+12 | 6637565 | 636 | 0.000000 | 1,48085E-08 |
| 0,613248 | 2,22955E+12 | 4868026 | 724 | 0.000000 | 1,28627E-08 |
| 0,645651 | 3,65382E+12 | 7031811 | 679 | 0.000000 | 4,6694E-09 |
| 0,604231 | 3,4863E+12 | 6857533 | 724 | 0.000000 | 2,85619E-09 |
| 0,585468 | 2,24291E+12 | 7358174 | 838 | 0.000000 | 7,52113E-10 |
| 0,595178 | 3,23276E+12 | 8296140 | 868 | 0.000000 | 9,02884E-10 |
| 0,625404 | 6,2379E+12 | 7514175 | 786 | 0.000000 | 8,10796E-09 |
| 0,637046 | 2,77043E+12 | 4906537 | 746 | 0.000000 | 1,77057E-09 |
| 0,642221 | 4,34443E+12 | 5369972 | 716 | 0.000000 | 1,20227E-08 |
| 0,600876 | 7,96383E+12 | 8822237 | 684 | 0.000000 | 1,16913E-08 |
| 0,625538 | 1,54241E+12 | 2501244 | 634 | 0.000000 | 3,91049E-09 |
| 0,591938 | 8,99869E+12 | 5555999 | 520 | 0.000000 | 6,97313E-08 |
| 0,624434 | 5,30348E+12 | 4157208 | 570 | 0.000000 | 2,23277E-08 |
| 0,662472 | 2,68201E+13 | 11700756 | 516 | 0.000000 | 3,74836E-07 |
| 0,636667 | 1,87834E+13 | 12176593 | 465 | -0.050000 | 1,43752E-06 |
| 0,560067 | 6,71415E+13 | 30933311 | 794 | -0.050000 | 1,70804E-07 |

| **Δ C terminus** | | | | | |
| --- | --- | --- | --- | --- | --- |
| **Shapiro Wilcox test of Surface factor measurements = 0,0000190864** | | | | | |
| **Surface factor** | **Total array intensity** | **Volume** | **Gain** | **Offset** | **Relative normalised intensity** |
| 0,608938 | 3,64178E+12 | 5593204 | 700 | -0.045000 | 3,0378E-08 |
| 0,636693 | 8,16335E+11 | 1201668 | 595 | -0.040000 | 3,9244E-09 |
| 0,602875 | 4,15393E+12 | 5680017 | 755 | -0.040000 | 6,66435E-09 |
| 0,591058 | 7,29635E+12 | 7264834 | 552 | -0.040000 | 4,13308E-07 |
| 0,615298 | 4,45103E+12 | 2971859 | 575 | -0.025000 | 2,70729E-07 |
| 0,652587 | 1,8507E+13 | 13450713 | 635 | -0.025000 | 1,90649E-07 |
| 0,626822 | 1,11982E+12 | 1543043 | 620 | -0.025000 | 3,54105E-08 |
| 0,511409 | 9,28248E+13 | 65081948 | 573 | -0.025000 | 1,90792E-06 |
| 0,628837 | 6,14053E+12 | 5200916 | 697 | 0.000000 | 4,87739E-08 |
| 0,635866 | 4,60462E+12 | 3255794 | 697 | 0.000000 | 3,62957E-08 |
| 0,5571 | 1,31272E+12 | 3757088 | 1152 | 0.000000 | 5,14308E-10 |
| 0,607552 | 9,62246E+12 | 12258184 | 695 | 0.000000 | 5,11426E-08 |
| 0,438512 | 8,1158E+12 | 11644622 | 1022 | 0.000000 | 3,32869E-09 |
| 0,639871 | 4,83877E+12 | 4136321 | 798 | 0.000000 | 4,11744E-08 |
| 0,632846 | 2,31286E+12 | 3016244 | 595 | 0.000000 | 6,45377E-08 |
| 0,64017 | 1,21844E+13 | 4738787 | 478 | 0.000000 | 1,60727E-06 |
| 0,582793 | 3,26088E+12 | 3970529 | 753 | 0.000000 | 2,59651E-08 |
| 0,602526 | 9,68692E+12 | 6884295 | 843 | 0.000000 | 8,51931E-09 |
| 0,605277 | 5,98395E+12 | 6994606 | 913 | 0.000000 | 7,73789E-09 |
| 0,603736 | 4,64526E+12 | 7211963 | 933 | 0.000000 | 2,18227E-09 |
| 0,606406 | 1,46181E+13 | 13429173 | 687 | 0.000000 | 8,24786E-08 |
| 0,626253 | 1,02688E+12 | 1249969 | 697 | 0.000000 | 1,14424E-08 |
| 0,642408 | 2,24659E+12 | 2841967 | 610 | 0.000000 | 4,65998E-08 |
| 0,638992 | 2,45641E+12 | 3766878 | 750 | 0.000000 | 7,52472E-09 |
| 0,652033 | 2,72134E+12 | 3969223 | 890 | 0.000000 | 2,49573E-09 |
| 0,624556 | 3,77378E+12 | 5086689 | 790 | 0.000000 | 1,66337E-08 |
| 0,622107 | 4,06591E+12 | 5914998 | 930 | 0.000000 | 3,76928E-09 |
| 0,625189 | 2,64048E+12 | 3996638 | 1020 | 0.000000 | 1,87336E-09 |
| 0,654479 | 4,41119E+12 | 4597798 | 662 | 0.000000 | 5,90209E-08 |
| 0,643535 | 3,37937E+12 | 2054127 | 689 | 0.000000 | 2,40121E-08 |
| 0,636256 | 1,67857E+12 | 2315217 | 589 | 0.000000 | 4,29122E-08 |
| 0,534767 | 3,24643E+12 | 4740745 | 739 | 0.000000 | 1,31906E-08 |
| 0,657605 | 4,5887E+12 | 5102355 | 587 | 0.000000 | 1,36971E-07 |
| 0,57229 | 1,22402E+13 | 15287482 | 642 | 0.000000 | 1,17108E-07 |
| 0,590369 | 2,82622E+12 | 4700929 | 732 | 0.000000 | 1,65038E-08 |

| **TRD** | | | | | |
| --- | --- | --- | --- | --- | --- |
| **Shapiro Wilcox test of Surface factor measurements = 0,00669844** | | | | | |
| **Surface factor** | **Total array intensity** | **Volume** | **Gain** | **Offset** | **Relative normalised intensity** |
| 0,598176 | 3,16893E+12 | 3502525 | 700 | 0.000000 | 2,14154E-08 |
| 0,625162 | 2,73209E+12 | 4127836 | 858 | 0.000000 | 6,41461E-09 |
| 0,634018 | 1,11616E+13 | 5618661 | 608 | 0.000000 | 2,31729E-07 |
| 0,643193 | 1,45012E+13 | 6331437 | 513 | 0.000000 | 1,17969E-06 |
| 0,595171 | 4,22043E+12 | 5171544 | 894 | 0.000000 | 6,38859E-09 |
| 0,641142 | 3,81971E+12 | 4553413 | 694 | 0.000000 | 1,69054E-08 |
| 0,603102 | 4,44032E+12 | 6155854 | 849 | 0.000000 | 6,8343E-09 |
| 0,610502 | 5,58117E+12 | 6803357 | 944 | 0.000000 | 1,82053E-09 |
| 0,63854 | 4,89811E+12 | 4076923 | 634 | 0.000000 | 6,19771E-08 |
| 0,552063 | 3,87515E+12 | 5715917 | 1012 | 0.000000 | 1,06057E-09 |
| 0,616207 | 3,27762E+12 | 3573019 | 748 | 0.000000 | 1,72601E-08 |
| 0,632833 | 5,37958E+12 | 4248590 | 543 | 0.000000 | 3,06982E-07 |
| 0,535099 | 1,8661E+13 | 28864171 | 690 | -0.045000 | 6,73705E-08 |
| 0,617852 | 2,10115E+12 | 4246632 | 705 | -0.045000 | 9,88302E-09 |
| 0,618984 | 2,59357E+12 | 1886377 | 690 | -0.045000 | 1,02208E-08 |
| 0,632971 | 1,08894E+13 | 5813173 | 475 | -0.025000 | 1,498E-06 |
| 0,620852 | 2,57747E+12 | 3352398 | 655 | -0.025000 | 4,3493E-08 |
| 0,602917 | 3,87134E+12 | 5693724 | 750 | -0.025000 | 1,02334E-08 |
| 0,591516 | 3,89636E+12 | 6193712 | 750 | -0.025000 | 1,13162E-08 |
| 0,572809 | 3,7833E+12 | 8853568 | 760 | -0.025000 | 1,40908E-08 |
| 0,557426 | 9,73109E+11 | 1920318 | 640 | -0.025000 | 1,28076E-08 |
| 0,533343 | 1,08255E+13 | 11142023 | 591 | -0.025000 | 1,32492E-07 |
| 0,60384 | 1,50659E+12 | 2413126 | 591 | -0.025000 | 2,67625E-08 |
| 0,533933 | 8,27921E+12 | 13419382 | 691 | -0.025000 | 2,23378E-08 |
| 0,632698 | 6,61901E+12 | 5161100 | 526 | -0.015000 | 6,46258E-07 |
| 0,60703 | 3,07537E+12 | 3160497 | 661 | -0.015000 | 3,06957E-08 |
| 0,566778 | 1,1543E+13 | 26238910 | 700 | -0.045000 | 3,23852E-08 |

| **C-terminus** | | | | | |
| --- | --- | --- | --- | --- | --- |
| **Shapiro Wilcox test of Surface factor measurements = 0,000664982** | | | | | |
| **Surface factor** | **Total array intensity** | **Volume** | **Gain** | **Offset** | **Relative normalised intensity** |
| 0,635374 | 1,93479E+13 | 9525875 | 500 | -0.060000 | 2,36012E-06 |
| 0,644299 | 2,8294E+12 | 2331535 | 545 | -0.060000 | 1,91073E-07 |
| 0,620814 | 6,23588E+12 | 8093795 | 590 | -0.060000 | 1,92457E-07 |
| 0,561285 | 3,98506E+13 | 29952265 | 506 | -0.060000 | 3,01329E-07 |
| 0,621814 | 1,7086E+13 | 10151186 | 481 | -0.060000 | 2,19326E-06 |
| 0,653822 | 1,82007E+13 | 13003596 | 486 | -0.060000 | 2,45022E-06 |
| 0,54875 | 1,34436E+13 | 7875785 | 551 | -0.060000 | 8,39183E-07 |
| 0,446414 | 5,04502E+12 | 15705879 | 684 | -0.060000 | 3,99999E-08 |
| 0,611969 | 1,39285E+13 | 14793369 | 594 | -0.055000 | 1,47181E-07 |
| 0,573015 | 8,84039E+12 | 19259969 | 614 | -0.055000 | 1,43474E-07 |
| 0,625504 | 9,28338E+12 | 14469617 | 659 | -0.055000 | 1,03453E-07 |
| 0,628427 | 3,27876E+12 | 6743959 | 649 | -0.055000 | 9,0456E-08 |
| 0,446022 | 1,11079E+13 | 18603980 | 559 | -0.055000 | 7,5234E-07 |
| 0,415884 | 9,53026E+13 | 39041466 | 539 | -0.055000 | 4,92035E-07 |
| 0,639145 | 6,40286E+12 | 5103660 | 562 | -0.045000 | 3,45463E-07 |
| 0,613603 | 3,42315E+12 | 6567071 | 817 | -0.045000 | 9,97214E-09 |
| 0,638109 | 2,04144E+12 | 3893507 | 712 | -0.045000 | 2,29411E-08 |
| 0,633211 | 2,03108E+12 | 3616099 | 682 | -0.045000 | 3,21347E-08 |
| 0,62632 | 7,44839E+12 | 6409764 | 517 | -0.045000 | 5,13908E-07 |
| 0,55112 | 5,4393E+12 | 6906488 | 651 | -0.045000 | 6,58339E-08 |
| 0,534472 | 3,03701E+13 | 17182344 | 654 | -0.045000 | 2,57888E-07 |
| 0,593064 | 2,88211E+12 | 8065728 | 751 | -0.045000 | 1,1165E-08 |
| 0,510987 | 7,29043E+12 | 14102133 | 746 | -0.045000 | 3,18628E-08 |
| 0,631565 | 1,66578E+13 | 9168834 | 858 | -0.045000 | 3,04339E-08 |
| 0,628529 | 2,76495E+12 | 4464642 | 721 | -0.045000 | 2,37293E-08 |
| 0,567224 | 9,31383E+12 | 17743035 | 848 | -0.045000 | 9,59877E-09 |
| 0,490004 | 7,96633E+12 | 14898458 | 700 | -0.045000 | 5,57617E-08 |
| 0,642699 | 4,10002E+13 | 20391143 | 770 | -0.045000 | 1,91819E-07 |
| 0,554087 | 5,57045E+12 | 8643390 | 710 | -0.045000 | 4,68186E-08 |
| 0,453038 | 3,3683E+12 | 11128316 | 796 | -0.060000 | 1,31953E-08 |

| **R133C** | | | | | |
| --- | --- | --- | --- | --- | --- |
| **Shapiro Wilcox test of Surface factor measurements = 0,000327978** | | | | | |
| **Surface factor** | **Total array intensity** | **Volume** | **Gain** | **Offset** | **Relative normalised intensity** |
| 0,46518 | 3,08105E+12 | 13047981 | 923 | -0.070000 | 1,61228E-09 |
| 0,614799 | 3,46887E+12 | 5247912 | 620 | -0.045000 | 5,53613E-08 |
| 0,628468 | 4,25124E+12 | 5025333 | 685 | -0.045000 | 2,6984E-08 |
| 0,619427 | 3,32569E+12 | 6592527 | 755 | -0.045000 | 2,25474E-08 |
| 0,618327 | 2,33463E+12 | 5519446 | 750 | -0.045000 | 1,51827E-09 |
| 0,601485 | 5,68681E+12 | 11606111 | 785 | -0.045000 | 1,89348E-08 |
| 0,430518 | 5,14503E+12 | 14644548 | 760 | -0.045000 | 1,06765E-08 |
| 0,43723 | 1,45477E+13 | 19629412 | 509 | -0.045000 | 1,22939E-06 |
| 0,605041 | 4,67245E+12 | 4403286 | 594 | -0.045000 | 8,19684E-08 |
| 0,524788 | 6,99668E+12 | 7087293 | 514 | -0.045000 | 5,92228E-07 |
| 0,438765 | 3,30922E+13 | 41898446 | 634 | -0.045000 | 6,25802E-07 |
| 0,634157 | 7,28532E+12 | 7031158 | 644 | -0.045000 | 7,49179E-08 |
| 0,593972 | 2,40038E+13 | 13365206 | 578 | -0.045000 | 2,22035E-07 |
| 0,612747 | 1,9701E+12 | 2985566 | 708 | -0.045000 | 1,05068E-08 |
| 0,45103 | 3,33151E+12 | 7289638 | 770 | -0.045000 | 3,9224E-09 |
| 0,518529 | 2,28856E+12 | 5872571 | 659 | -0.040000 | 1,11253E-08 |
| 0,598976 | 4,35137E+12 | 7142122 | 701 | -0.040000 | 3,96128E-08 |
| 0,622977 | 2,67729E+12 | 5548166 | 848 | 0.000000 | 3,58667E-09 |
| 0,602327 | 1,72168E+13 | 15980677 | 612 | 0.000000 | 7,04093E-08 |
| 0,636445 | 1,03918E+13 | 10628981 | 757 | 0.000000 | 7,44656E-09 |
| 0,578103 | 6,38761E+12 | 8358802 | 842 | 0.000000 | 2,34722E-09 |
| 0,522258 | 9,48996E+12 | 14726139 | 842 | 0.000000 | 3,45199E-09 |
| 0,315381 | 4,77525E+12 | 6802052 | 742 | 0.000000 | 2,83363E-09 |
| 0,632288 | 6,11203E+12 | 4702887 | 620 | 0.000000 | 1,63E-08 |
| 0,608009 | 5,8098E+12 | 6458065 | 797 | 0.000000 | 2,53136E-09 |
| 0,560548 | 7,45251E+12 | 7587280 | 797 | 0.000000 | 3,33145E-09 |
| 0,64087 | 8,56955E+12 | 5349738 | 767 | 0.000000 | 5,01064E-09 |
| 0,568854 | 1,10507E+13 | 13997044 | 971 | 0.000000 | 1,06372E-09 |
| 0,50306 | 4,05403E+13 | 23795758 | 700 | 0.000000 | 3,36215E-08 |
